# Nucleophilic Covalent Ligands Enable Simultaneous Surface Reconstruction and Passivation of Colloidal InSb Quantum Dots for Stable Short‐Wave Infrared Photodetectors

**DOI:** 10.1002/anie.202505179

**Published:** 2025-05-22

**Authors:** Yangning Zhang, Muhammad Imran, Pan Xia, Yiqing Chen, Ahmet Gulsaran, Yanjiang Liu, Ehsan Nikbin, Benjamin Rehl, Lizhou Fan, Filip Dinic, Da Bin Kim, Lewei Zeng, Mustafa Yavuz, Sjoerd Hoogland, Edward H. Sargent

**Affiliations:** ^1^ Department of Electrical and Computer Engineering University of Toronto 10 King's College Road Toronto Ontario M5S 3G4 Canada; ^2^ Waterloo Institute for Nanotechnology, Department of Mechanical and Mechatronics Engineering University of Waterloo 200 University Ave W Waterloo Ontario N2L 3G1 Canada; ^3^ Department of Materials Science and Engineering University of Toronto 184 College St Toronto Ontario M5S 3E4 Canada

**Keywords:** Colloidal quantum dots, Infrared photodetectors, Stability, Surface passivation, Surface reconstruction

## Abstract

Indium antimonide (InSb) colloidal quantum dots (CQDs) are promising candidates for short‐wave infrared (SWIR) photodetectors due to their large Bohr exciton radius and tunable bandgap in the 0.6–1.3 eV range. However, the formation of metal oxides on InSb surfaces during synthesis impedes charge transport, necessitating CQD resurfacing strategies for integration into photodetectors. Previous reports achieved progress in device efficiency by resurfacing these CQDs with acid‐halide sequential treatments, but the device operating stability remains unsatisfactory. Herein, we report a solution‐phase strategy for surface reconstruction and passivation of InSb CQDs using sulfur‐based nucleophilic covalent ligands. We find that short‐chain thiol molecules remove surface metal oxides through nucleophilic attack and enable robust passivation of In and Sb via strong covalent bonds, whereas metal sulfides are less effective at oxide removal and passivation. Consequently, the thiolate‐passivated CQDs exhibit a tenfold decrease in trap state density compared to controls and remain structurally and optically stable for 5 months. We demonstrate InSb CQD SWIR photodetectors that realize a high external quantum efficiency (EQE) of 28% at 1450 nm, with the highest operating stability among reported CQD SWIR photodetectors, retaining 95% of performance following 300 h of biased and illuminated operation.

## Graphical abstract

A simultaneous surface reconstruction and passivation strategy is designed for indium antimonide (InSb) colloidal quantum dots (CQDs) using sulfur‐based nucleophilic covalent ligands. This leads to stable CQD inks and 1450 nm‐operating InSb CQD photodetectors with 28% external quantum efficiency and 300 h operating lifetime.

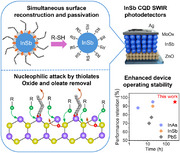

## Introduction

Colloidal quantum dots (CQDs) are attractive materials for next‐generation infrared photodetectors because of their tunable bandgaps, solution‐processability, and potential for low‐cost production.^[^ ^1^, ^2^, ^3^ ^]^ CQD photodetectors operating in the short‐wave infrared (SWIR, 1000–2500 nm) range are particularly relevant to a range of applications including augmented reality, authentication, telecommunications, surveillance, medical diagnostics, and environmental monitoring.^[^ ^4^, ^5^, ^6^ ^]^

To date, most high‐performance CQD photodetectors rely on Pb‐ and Hg‐containing CQDs as the absorbing layer,^[^ ^3^, ^7^, ^8^, ^9^ ^]^ and these elements are (along with Cd) regulated by the Restriction of Hazardous Substances (RoHS) directive. For this reason, RoHS‐compliant CQDs, such as InSb,^[^ ^10^, ^11^ ^]^ InAs,^[^ ^12^, ^13^, ^14^ ^]^ and Ag_2_Te,^[^ ^15^, ^16^, ^17^ ^]^ are seeing intensive exploration. Indium antimonide (InSb), with a large Bohr exciton radius of ∼60 nm and a direct bulk bandgap of 0.17 eV,^[^ ^18^ ^]^ allows for quantum‐size tuning of the bandgap at relatively modest particle sizes. Recent advances in the synthesis of InSb CQDs, such as the diffusion‐controlled growth approaches, have enabled more precise control of the CQD size and, hence, their bandgap.^[^ ^11^, ^19^, ^20^, ^21^ ^]^ The ability to tune InSb CQDs’ bandgap in the range of 0.6 to 1.3 eV expands their potential application for heavy‐metal‐free SWIR photodetectors.

Given the oxophilic nature of In and Sb and the relatively weak In─Sb bonds, InSb CQDs are more susceptible to oxidation compared to other SWIR‐active CQDs, including PbS or InAs.^[^ ^22^, ^23^, ^24^ ^]^ In and Sb oxides are easily formed on the CQD surfaces during synthesis, which is known to militate against the needed efficient charge transport. In addition, oxidation‐induced structural distortions lead to trap states within the bandgap.^[^ ^10^, ^25^ ^]^ Therefore, removing the native metal oxides and resurfacing InSb CQDs for efficient charge transport is one of the main challenges for their integration into high performance photodetectors.

Surface chemistry plays a critical role in tuning both the electronic properties and stability of CQDs and directly influences the efficiency and durability of resulting devices. Recent studies have utilized acidic ligands to remove metal oxides from InSb CQD surfaces, followed by halide treatment for passivation and conductivity enhancement.^[^ ^10^, ^11^ ^]^ While these sequential strategies have led to improvements in device efficiency, the photodetectors have so far exhibited limited operating stability. With Time‐of‐Flight Secondary Ion Mass Spectrometry (ToF‐SIMS) studies, we observed iodine ion migration from the InSb layer to the Ag electrode, which is likely the cause of device performance degradation (Figure S1). In addition, prior InSb CQD resurfacing approaches mostly utilize solid‐state ligand exchange processes, which are known for limited control over ligand coverage and film homogeneity.^[^ ^26^ ^]^

Herein, we pursue a solution‐phase surface reconstruction and passivation strategy for InSb CQDs using sulfur‐based nucleophilic covalent ligands, aiming to ensure the device efficiency united with operating stability. Density functional theory (DFT) simulations suggest that sulfur‐based ligands form strong bonds with InSb CQD surfaces. We experimentally investigate the chemical interactions between these ligands and metal oxides on InSb CQD surfaces, finding that thiols, particularly those with reduced steric hindrance, are more effective in removing metal oxides via nucleophilic attack, facilitating simultaneous passivation of surface In and Sb atoms. In contrast, metal sulfides exhibit limited reactivity toward surface oxides due to solvation effects, leading to inadequate passivation. Specifically, the mercaptoethanol (ME)‐passivated CQDs exhibit a 10‐fold decrease in trap state density compared to the sulfide‐treated ones and maintain their structural integrity, colloidal stability, and optical absorption for at least 5 months. Employing these resurfaced CQDs as the active layer, we fabricate InSb CQD SWIR photodetectors that achieve an external quantum efficiency (EQE) of 28% at 1450 nm. These devices preserve 95% of their initial performance after 300 h of continuous operation under bias and illumination, marking a 10‐fold improvement over previously reported halide‐passivated counterparts.

## Results and Discussion

InSb CQDs were synthesized following a hot injection method using InCl_3_ and SbCl_3_ as precursors and alane N,N‐dimethylethylamine as the reducing agent.^[^ ^27^ ^]^ ZnBr_2_ and trioctylamine were used as additives to control the reaction kinetics, which enables the uniform growth of CQDs with an average diameter of 5.1 nm (see Supporting Information for experimental details, and Figure S2 for transmission electron microscopy images). Oleic acid was added prior to the purification of these CQDs to enhance their colloidal stability in nonpolar solvents. XPS analysis of as‐prepared InSb CQDs (i.e., OA‐capped CQDs) indicates that they are coated with surface layers of In_2_O_3_ and Sb_2_O_x_ (*x* = 3 or 5) along with In‐oleate (Figure S3). DFT simulations suggested that the metal‐oxygen bonds induce local structural distortions, leading to the formation of trap states within the bandgap.^[^ ^10^ ^]^ Furthermore, the presence of surface metal oxides and In‐oleate species impedes charge transport between CQDs.

To remove native In‐oleate and metal oxides and to reduce surface defects, we design a simultaneous surface reconstruction and passivation (SSRP) strategy using sulfur‐containing ligands. Sulfur exhibits stronger nucleophilicity than oxygen due to its higher polarizability and more diffuse valence orbitals. The strong nucleophilicity enables sulfur‐based reagents to attack electrophilic metal centers coordinated to surface oxides, displacing oxygen and forming metal─sulfur bonds (similar to the nucleophilic substitution reactions in organic chemistry).^[^ ^28^ ^]^ DFT simulations suggest that sulfur‐based ligands, including organic thiol molecules or inorganic metal sulfides, bind more strongly to the surface of InSb CQDs compared to halide ligands (Figure S4), suggesting that they could counter ligand desorption and migration, thus enhancing InSb CQD stability and device stability.

The nucleophilic reactivity of sulfur‐based ligands is influenced by solvation effects and local chemical environment of electrophilic sites. Thiols, typically solubilized in aprotic solvents like DMF, retain their strong reactivity as their nucleophilic centers have minimal interaction with the solvents. In nucleophilic reactions involving oleate‐coated surfaces with steric hindrance, short‐chain thiols are expected to be more effective as the smaller molecular size enables them to weave through oleate ligands to reach and bond with metal sites. Metal sulfides are less soluble in aprotic solvents but more soluble in protic solvent like formamide, which stabilizes the sulfide ions through hydrogen bonding. This solvation effect restricts the interaction between the sulfide ions and the metal atoms and slows down the reaction kinetics.

**Figure 1:**
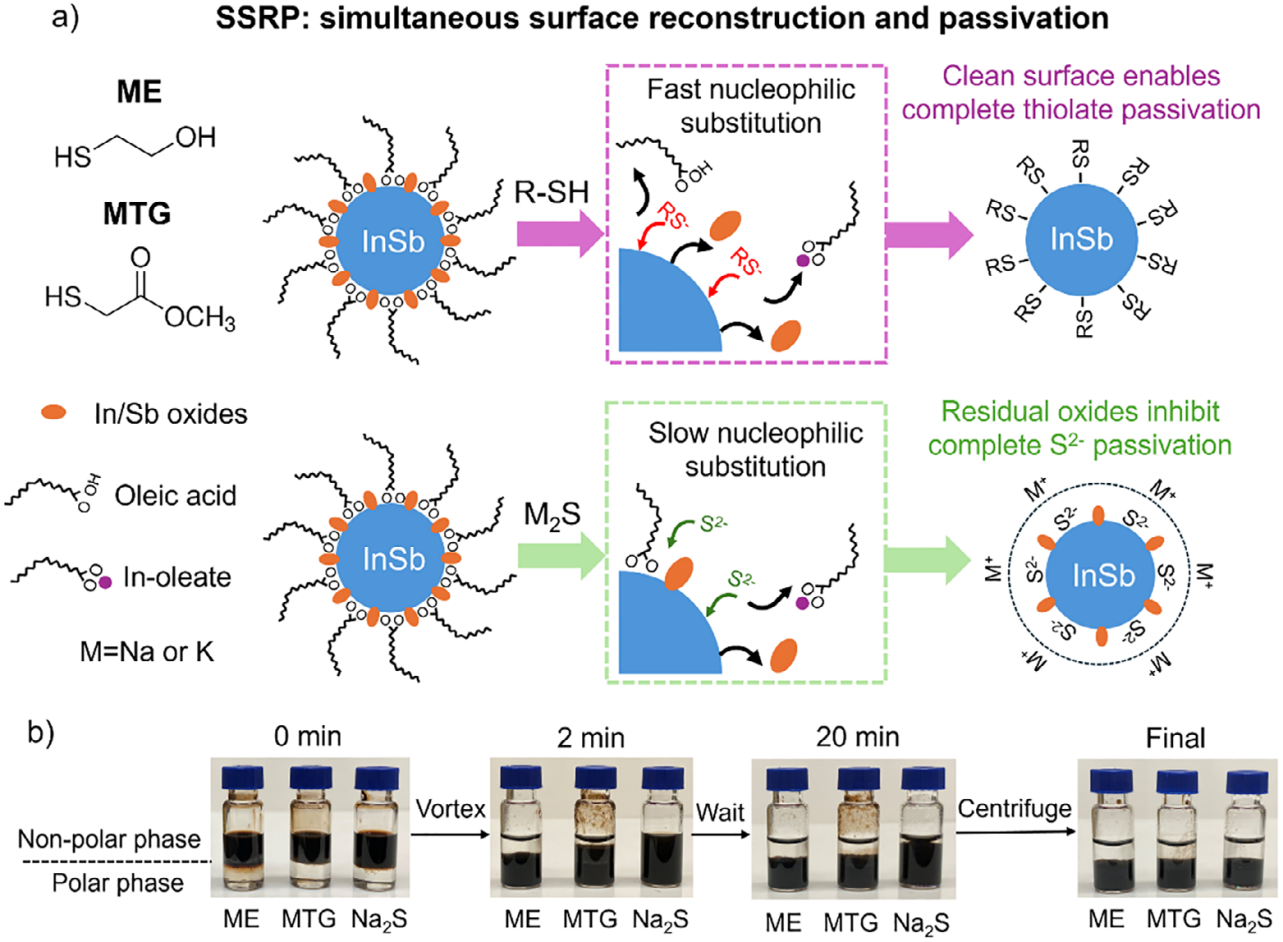
a) Schematic illustration of simultaneous surface reconstruction and passivation strategy for InSb CQDs. b) Photographs taken during the two‐phase ligand exchange processes.

As shown in the schematic illustration (Figure 1a), treatment with short‐chain thiols cleans the CQD surface by rapidly removing both the surface metal oxides and oleate ligands through strong nucleophilic attack. The resulting oxide‐free and oleate‐free CQD surface is then passivated by thiolates. In contrast, treatment with metal sulfides features a slow displacement of oleate ligands, hindered by the limited access of S^2‐^ ions to the metal sites due to solvation effects. This process leaves behind oxide residues, which block certain surface sites and limit the attachment of S^2‐^ ions, leading to incomplete passivation of the CQDs.

We considered two key factors when selecting thiol ligands: First, the organic backbone should be minimized not only to facilitate access to metal centers but also to reduce the distance between CQDs, thereby enhancing charge transport. Second, the molecules should possess a secondary functional group that exhibits some affinity for polar aprotic solvents, needed to enable CQD dispersibility.^[^ ^29^ ^]^ Suitable candidates include 2‐mercaptoethanol (ME) containing a hydroxyl group and methyl thioglycolate (MTG) containing an ester group.

We then pursued two‐phase solution ligand exchange processes to replace the native oleate ligands with ME, MTG, and sodium sulfide (Na_2_S). The new ligands dissolved in polar solvents were added to OA‐capped InSb CQDs dispersed in a nonpolar solvent. There was a clear separation between the top nonpolar phase and the bottom polar phase (see photographs in Figure 1b). This two‐phase solution mixture was then vortexed vigorously until the CQDs completely transferred from the nonpolar phase to the polar phase, indicative of ligand replacement (see Supporting Information for experimental details). We observed 10x faster phase transfer kinetics for ME and MTG ligands over Na_2_S ligands, suggesting faster replacement of oleate ligands by the thiolates. The slow exchange kinetics of Na_2_S ligands indicate that solvated S^2‐^ ions have difficulty accessing metal sites, which are sterically hindered by oleate ligands. After the oleate ligands are mostly removed, the negatively charged S^2‐^ ions are bound to the CQDs and provide electrostatic stabilization.^[^ ^30^ ^]^

**Figure 2:**
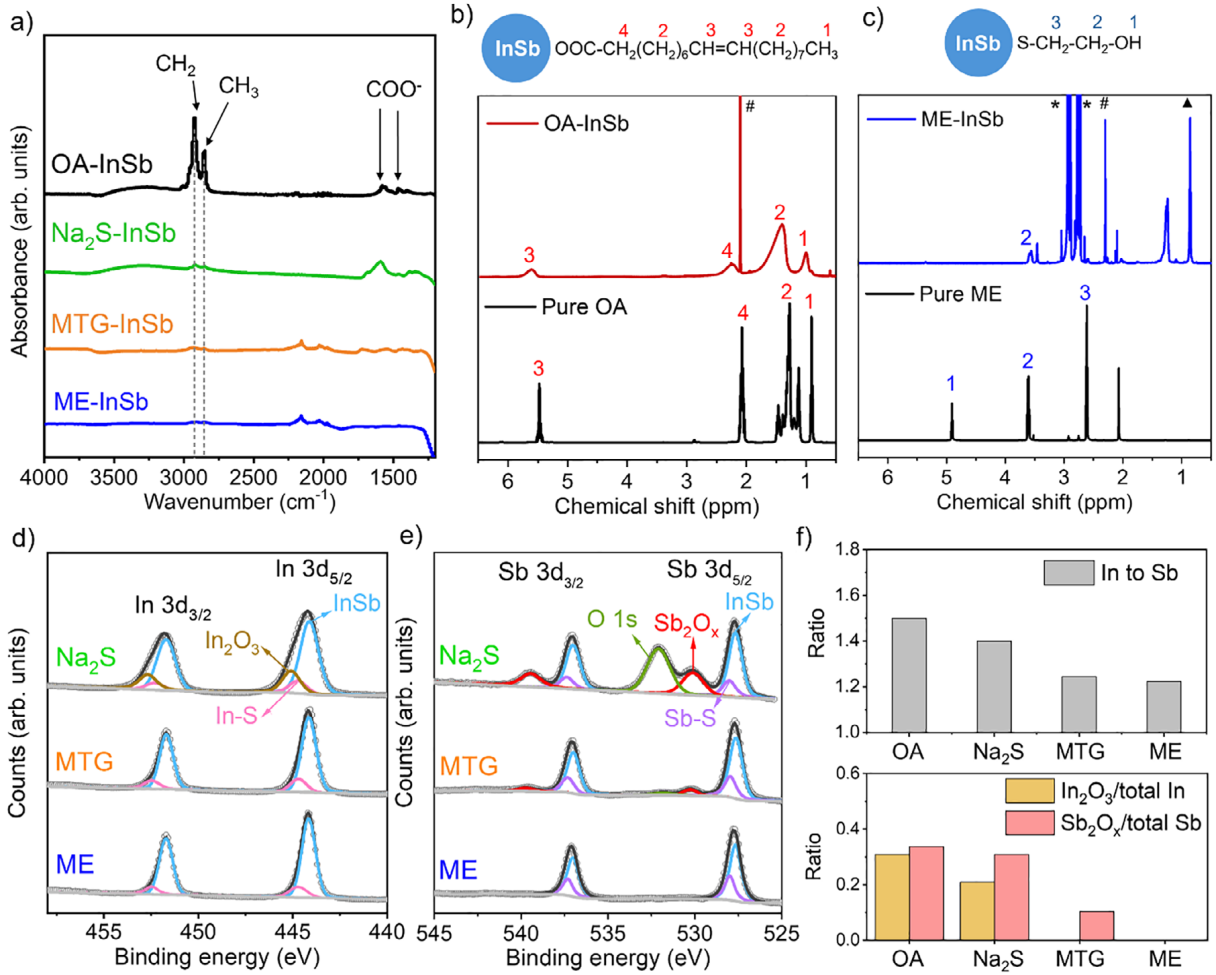
a) FTIR spectra of OA‐capped CQDs and exchanged CQDs. ^1^H‐NMR spectra of b) OA‐capped InSb CQDs and pure OA ligands, c) ME‐exchanged InSb CQDs and pure ME ligands. Signals from residual solvents including toluene, hexane, and DMF are marked with #, ▲, and *, respectively. XPS spectra of CQDs after different surface modifications: d) In 3d spectra; e) Sb 3d spectra; f) Calculated In to Sb atomic ratio, In_2_O_3_ to total In ratio, Sb_2_O_x_ to total Sb ratio.

Figure 2a shows Fourier transform infrared (FTIR) spectra before and after ligand exchange. The significant reduction of FTIR peaks at 2855 and 2923 cm^−1^ (corresponding to CH_3_ and CH_2_ stretches) in all the exchanged CQDs indicates that the original OA ligands have been substantially removed. For Na_2_S‐exchanged CQDs, the broad peak at 3320 cm^−1^ and the sharp peak at 1595 cm^−1^ are assigned to residual formamide solvent. In Figure 2b, the ^1^H‐Nuclear Magnetic Resonance (NMR) spectra of OA‐capped CQDs exhibit a characteristic peak at 5.5–5.6 ppm, corresponding to the *3*‐protons of the C═C bond in oleate ligands. To investigate the thiol ligand exchange process, we use ME‐exchanged CQDs as a model system to perform ^1^H‐NMR analysis (Figure 2c). In these CQDs, the characteristic oleate peaks are no longer observable, confirming the complete removal of oleate ligands. The broad peak at 3.5–3.6 ppm in the ME‐exchanged CQDs corresponds to the *2*‐protons of ME ligands, suggesting that ME ligands are bound to CQD surface. The ^1^H‐NMR spectrum of Na₂S‐treated CQDs also showed an absence of oleate protons (Figure S5). XPS spectra of S 2p in Figure S6 provide evidence for thiolate attachment to ME‐ or MTG‐treated CQDs and sulfide attachment to Na₂S‐treated CQDs.

The thiol‐mediated resurfacing strategy proves more efficient in oxide removal compared to metal sulfide treatment. Figure 2d,e show the XPS spectra of In 3d and Sb 3d for CQDs treated with different ligands. The calculated In to Sb atomic ratio, In_2_O_3_ to total In ratio, and Sb_2_O_x_ to total Sb ratio for each sample are summarized in Figure 2f. Notably, ME‐treated InSb CQDs were completely free of In_2_O_3_ and Sb_2_O*_x_*. In contrast, MTG‐treated InSb CQDs showed 10% Sb_2_O*_x_* in the Sb peaks, while the In spectra showed an absence of In_2_O_3_. Compared with MTG‐treated ones, Na_2_S‐treated CQDs contained three times more Sb_2_O_x_, and the In peaks consisted of 20% In_2_O_3_. Similarly, K_2_S‐exchanged CQDs exhibited a significant amount of remaining surface In/Sb oxides (Figure S7). Across all samples after thiol or sulfide treatment, In─S and Sb─S bonds were observed, with a higher prevalence in thiol‐treated CQDs than in those treated with sulfides, indicating superior passivation of surface In and Sb atoms by thiolates.

**Scheme 1:**
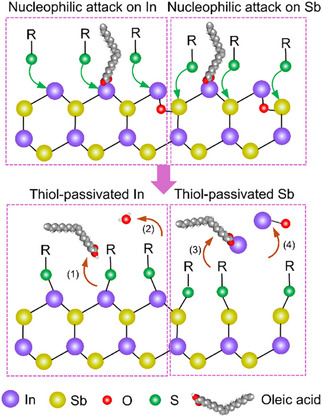
Illustration of thiol‐mediated surface reconstruction mechanism. Left: thiols attack the surface In atoms, leading to the detachment of (1) oleic acid and (2) O^2‐^ anions that combine with free protons to form water. Right: thiols attack the surface Sb atoms, releasing (3) In‐oleate and (4) In oxides.

The OA‐capped InSb CQDs are In‐rich, with an In to Sb ratio of 1.5. This ratio decreased to 1.2 after thiol treatment and 1.4 after Na_2_S treatment (Figure 2f). From this, we infer that the resurfacing process involves the detachment of some surface In atoms. XPS analysis of the dried supernatant collected during thiol treatment (Figure S8) revealed the presence of In_2_O_3_ and In‐oleate as by‐products. Interestingly, no Sb signal was detected in the supernatant, suggesting that the surface Sb atoms remain intact on the CQDs. We propose a few possible scenarios for oleate/oxide removal in Scheme 1: (1) Thiols nucleophilically attack In atoms bonded with oleate, resulting in the removal of oleic acid. (2) On In─O─Sb sites, thiols cleave the In─O and Sb─O bonds simultaneously, releasing oxygen anions that combine with protons to form water.^[^ ^31^ ^]^ Finally, thiols can also access Sb atoms and break In─Sb bonds due to their relatively weak bond dissociation energy.^[^ ^32^ ^]^ This would remove some of the surface In atoms as (3) In‐oleate and (4) In oxides. This thiol‐mediated SSRP process eliminates surface oxides and oleate species, allowing thiolates to passivate both surface In and Sb atoms. For Na_2_S treatment, the aforementioned solvation effect reduces their reactivity. Steric hindrance from the remaining oleate ligands further slows down the access of S^2^⁻ ions to the metal cations, which limits the efficiency of oxide removal. Consequently, some In/Sb oxides are left on the Na_2_S‐treated CQDs.

**Figure 3:**
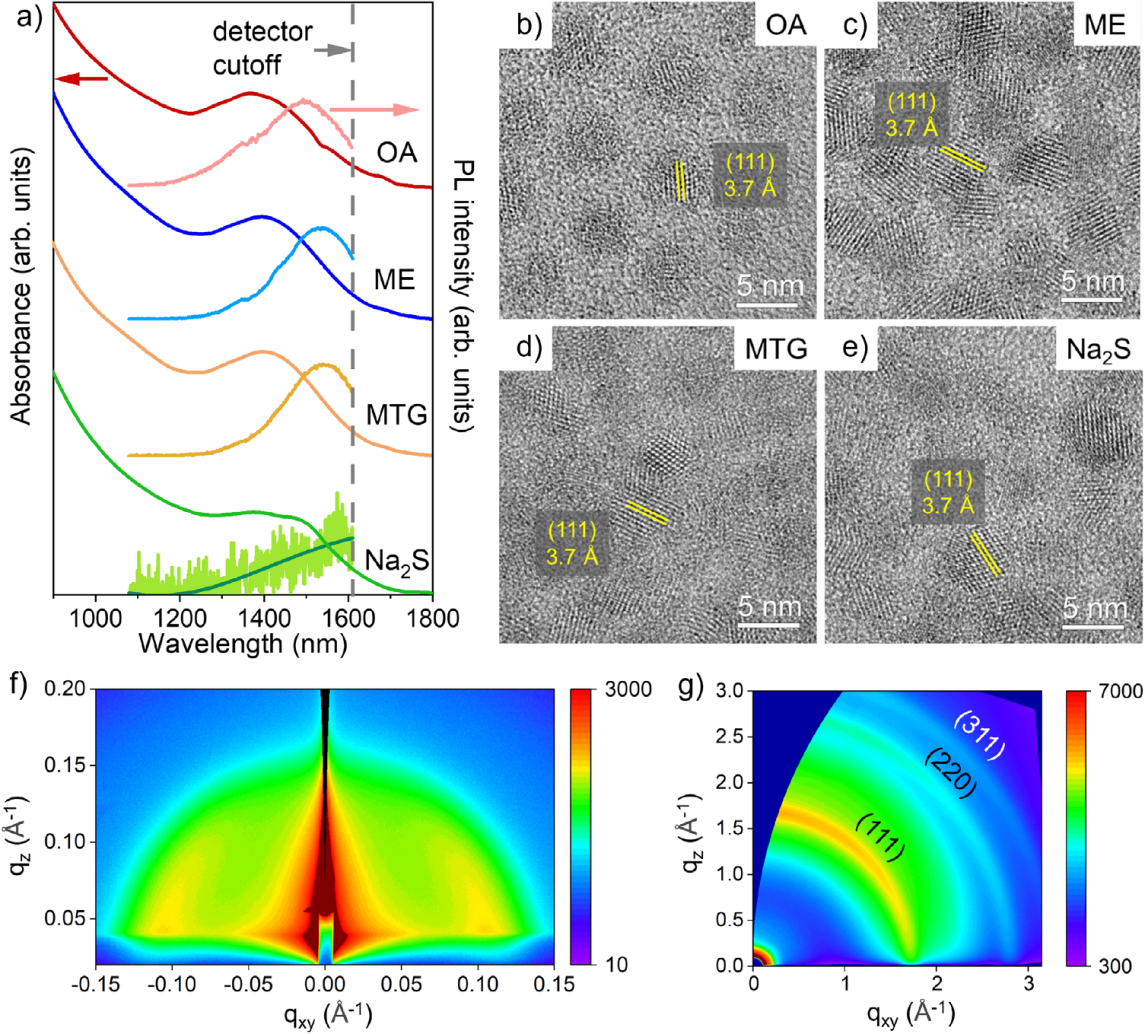
a) Absorbance and PL spectra, and b)–e) high‐resolution TEM images of InSb CQDs passivated with different ligands (OA, ME, MTG, or Na_2_S). The lattice spacings of 3.7 Å in (b)–(e) correspond to the (111) planes of InSb with zinc blende crystal structure. f) GISAXS and g) GIWAXS of ME‐passivated CQDs.

Steady‐state absorbance and photoluminescence (PL) spectra of ligand‐exchanged CQDs are compared in Figure 3a. ME‐ or MTG‐exchanged CQDs exhibited an absorbance excitonic peak at 1400 nm with a peak‐to‐valley ratio of 1.2. Although thiol treatment may slightly reduce the effective particle size through surface oxide removal, no blueshift is observed in the absorbance excitonic peak. Instead, a ∼20 nm redshift is observed compared to OA‐capped CQDs (Table S1). This redshift is attributed to partial delocalization of the electron‐hole wavefunction into the thiol ligands, which slightly narrows the CQD bandgap.^[^ ^33^ ^]^ The absorbance peak of Na_2_S‐exchanged CQDs showed a 3x larger redshift along with some broadening, suggesting more extensive wavefunction delocalization.^[^ ^30^ ^]^ The PL peaks of ME‐ or MTG‐exchanged CQDs were centered at around 1540 nm, respectively, with a Stokes shift of 140 nm and an estimated PL quantum yield (PL QY) of 0.5%. In contrast, Na_2_S exchange significantly quenched the PL of CQDs, with the peak center red‐shifted to >1600 nm. The Stokes shift of Na_2_S‐exchanged CQDs was at least 17% larger than that of thiol‐exchanged ones (Table S1). K_2_S‐exchanged CQDs also showed a very weak PL and a Stokes shift of more than 200 nm (Figure S7).

After treatment with the above‐mentioned thiols and sulfides, CQDs retained their original size and shape, as shown in the scanning transmission electron microscopy (STEM) images in Figure S9. ME‐InSb CQDs had the smallest interparticle distance (i.e., average CQD center‐to‐center distance) of 5.5 nm among all samples, which is a reduction of 35% compared to that of OA‐capped CQDs. The minimized spacing between CQDs after ligand exchange could enhance electronic coupling among CQDs and improve charge transport. The bulkier organic backbone of MTG ligands leads to a 5% increase in interparticle distance compared to ME‐InSb CQDs. Na_2_S‐InSb CQDs had the largest interparticle distance of 6.1 nm among all exchanged CQDs, due to the remaining native ligands and surface oxides. High‐resolution TEM images of OA‐capped CQDs (Figure 3b) show lattice fringes corresponding to the zinc blende phase of InSb, with no evidence of crystalline oxide phases, suggesting that the surface oxide layer is likely amorphous. However, atomic‐resolution imaging would be needed to distinguish between a continuous oxide shell or isolated oxide clusters. After thiol or sulfide treatment, CQDs retain the same crystal structure (Figure 3c–e). This is further supported by X‐ray diffraction (XRD, in Figure S10), which shows no additional peaks associated with crystalline In_2_O_3_ or Sb_2_O_x_, indicating that the CQDs maintain their phase purity throughout the treatment process.

We select ME‐InSb CQDs as a representative sample for grazing‐incidence small‐angle and wide‐angle X‐ray scattering (GISAXS/GIWAXS) measurements. GISAXS (Figure 3f) shows a sharp scattering peak with *q* of 1.16 nm^−1^. This corresponds to an average interparticle distance of 5.4 nm, which aligns closely with our measurement of 5.5 nm using STEM imaging. GIWAXS (Figure 3g) shows three characteristic peaks that are indexed to the (111), (220), and (311) lattice planes of the zinc blende crystal structure, consistent with XRD results.

**Figure 4:**
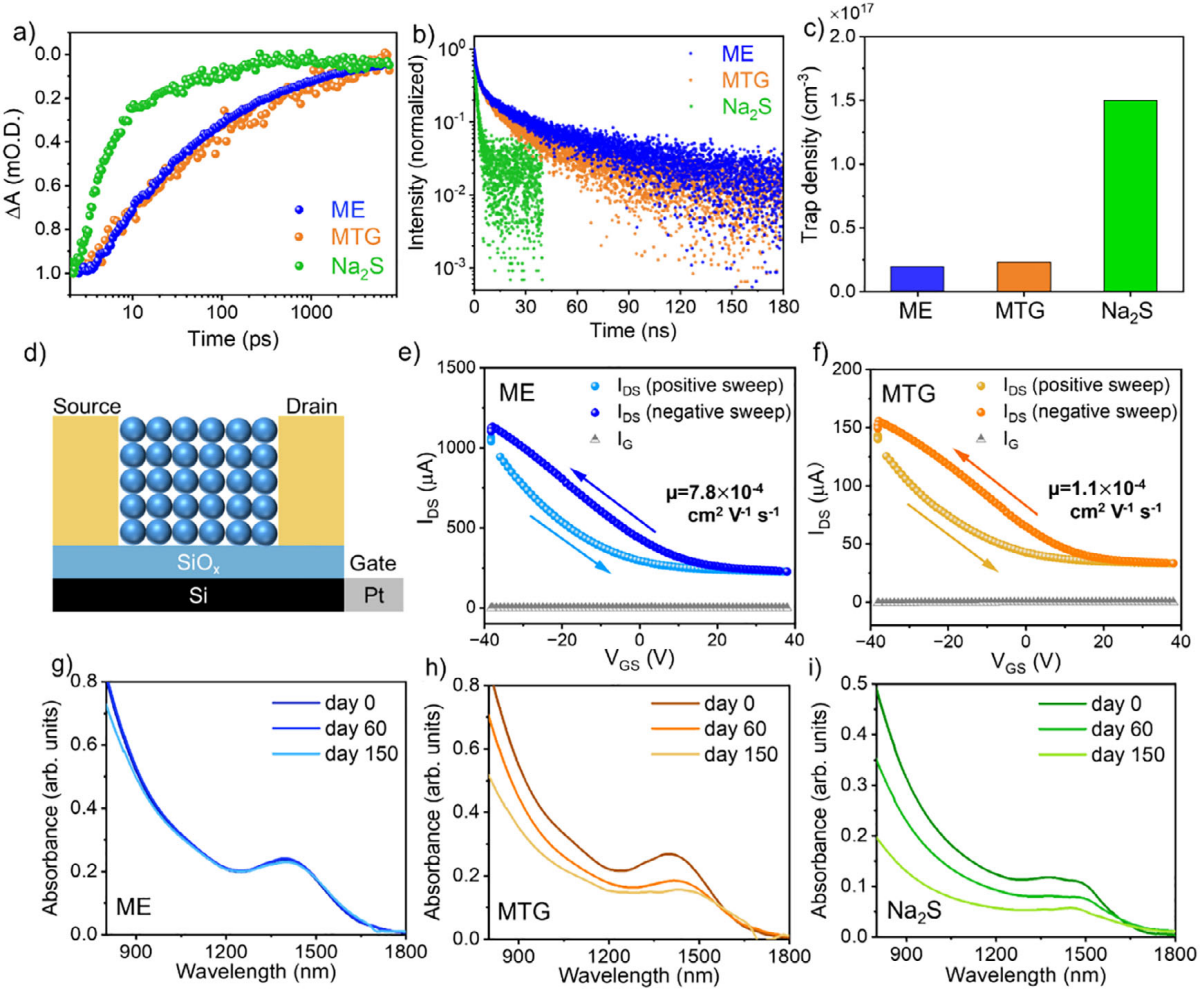
a) TA decay dynamics and b) time‐resolved PL of InSb CQDs passivated with different ligands (ME, MTG, or Na_2_S). c) Comparison of CQD trap densities determined from SCLC measurements. d) Schematic of field‐effect transistor (FET) device structure. Transfer curves of e) ME‐InSb and f) MTG‐InSb CQDs. Arrows show the sweeping directions. g)–i) Comparison of CQD ink stability: absorbance spectra of g) ME‐InSb, h) MTG‐InSb, and i) Na_2_S‐InSb CQDs before and after 150 days of storage.

Transient absorption (TA) and time‐resolved PL (TRPL) measurements were performed to understand the impact of surface chemistry on carrier dynamics (Figure 4a,b). The TA decay kinetics of ME‐ and MTG‐exchanged CQDs were very similar, with an average lifetime of 1.3–1.5 ns (see Figure S11 for the TA spectral kinetics and Table S2 for fitting parameters). Na_2_S‐exchanged CQDs had much faster exciton trapping and thus a faster recovery, with an average lifetime of <100 ps. The TRPL data was fitted with a three‐exponential decay model, with an average lifetime of 42 ns and 24 ns for ME‐ and MTG‐exchanged CQDs, respectively (see Table S3 for fitting parameters). The PL lifetime of Na_2_S‐exchanged CQDs appeared to be 10x shorter. The faster carrier dynamics, together with the larger Stokes shift, suggest higher trap state density in Na_2_S‐exchanged CQDs. This is attributed to the incomplete removal of surface oxides and inadequate surface passivation.

We estimated the trap state densities based on the trap‐filled regime in space‐charge limited current (SCLC) measurements (Figure S12 and Table S4). In Figure 4c, ME‐ and MTG‐exchanged CQDs showed one order of magnitude lower trap state densities than that of Na_2_S‐exchanged CQDs, supporting our speculations from transient optical measurements.

To investigate the charge transport properties of thiol‐ and sulfide‐exchanged InSb CQDs, we carried out field‐effect transistor (FET) measurements on CQD films (the FET device structure is shown in Figure 4d). In the transfer curves of ME‐ and MTG‐passivated CQDs (Figures 4e,f), the drain‐source current (I_DS_) increased with decreasing gate‐source voltages (V_GS_), suggesting the p‐type behavior of these CQDs. The output curves are provided in Figure S13. Na_2_S‐exchanged CQDs did not exhibit FET behavior, likely due to their high trap state density. The hole mobility of ME‐InSb CQDs was estimated to be 7.8 × 10^−4 ^cm^2^ V^−1^ s^−1^, which is seven times higher than that of MTG‐InSb CQDs (Table S5). The improved hole mobility of ME‐InSb CQDs results from their smaller interparticle spacing and complete removal of surface oxides, both of which enhance charge transport.

To compare the colloidal stability of exchanged CQDs, we monitored the absorbance spectra of exchanged CQD inks stored under nitrogen over 150 days (Figures 4g–i). To the best of our knowledge, the colloidal stability of previously reported III‐V CQDs utilizing halide passivation has been limited to 2 days.^[^ ^34^ ^]^ Notably, the optical absorption of ME‐passivated InSb CQDs after 150 days largely overlapped with that of fresh CQDs, showing no degradation of the excitonic peak. TEM further confirmed that their size, shape, and crystal structure remain unchanged (Figure S14). The superior colloidal stability of ME‐passivated CQDs indicates that the ME molecules bind strongly to CQD surfaces, and thus, ligand desorption is suppressed. In contrast, the absorbance peaks of MTG‐ and Na_2_S‐exchanged CQDs red‐shifted by 25 nm and 60 nm, respectively, accompanied by a 40–50% decrease in optical density. In both types of CQDs, aggregation was formed in the cuvettes due to ligand detachment from the CQD surfaces over time. Thus, MTG and S^2‐^ ligands are not robust enough to passivate InSb CQDs.

**Figure 5:**
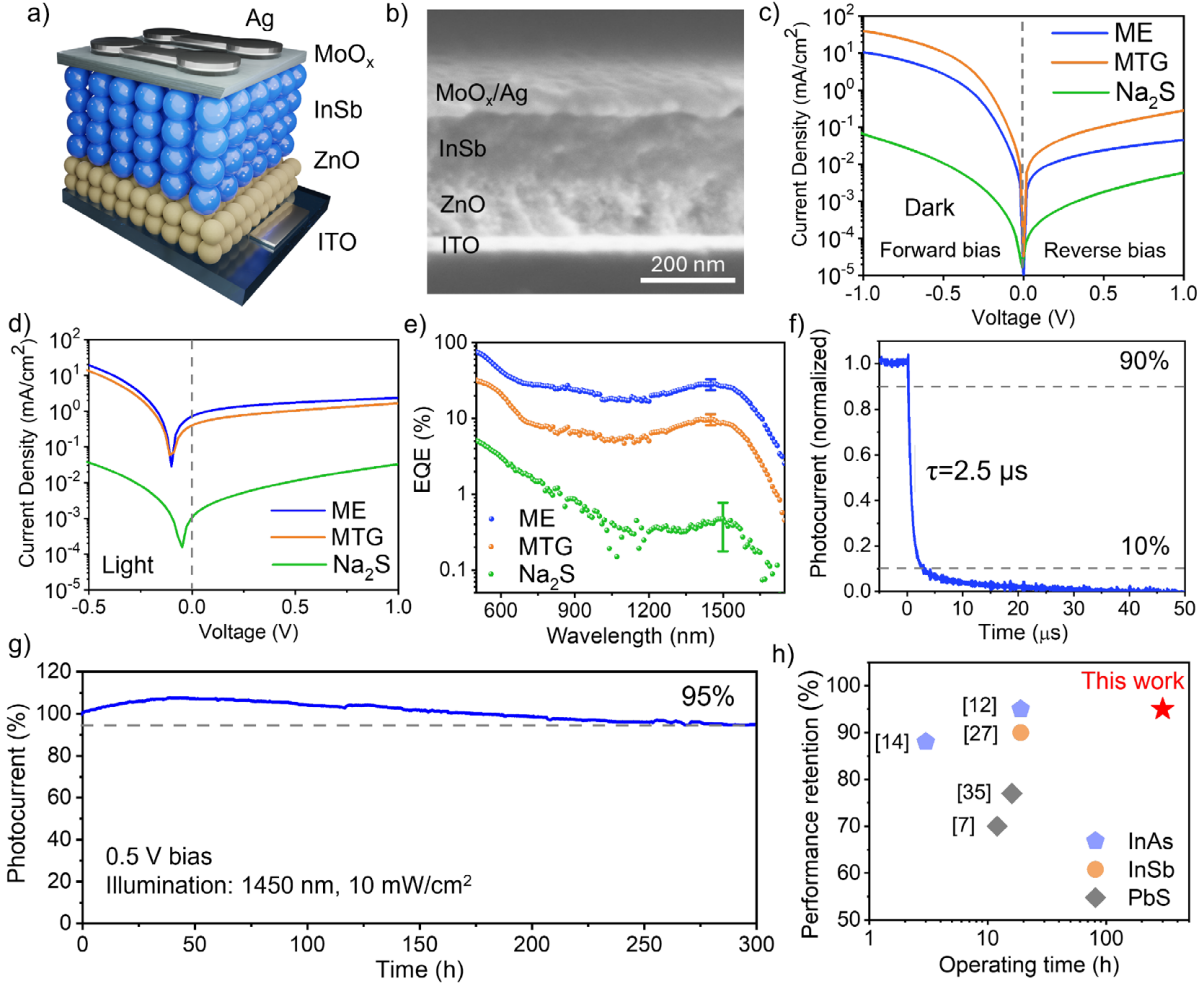
a) Schematic of InSb CQD photodetector device stack and b) cross‐sectional SEM image. Comparison of c) dark and d) photocurrent density‐voltage curves, and e) EQE spectra of photodetectors employing InSb CQDs passivated with different ligands. The error bars are based on standard deviation with a sample size of 5. f) Transient photocurrent and g) operating stability of ME‐passivated InSb CQD photodetectors. In (g), the slight increase in photocurrent in the first 50 h is attributed to the enhanced conductivity of CQDs under illumination‐induced local heating. h) Comparison of operating stability reported in this work with prior studies.

Using the InSb CQDs passivated with different ligands as the active layer, we fabricated SWIR photodetectors with the vertical architecture of ITO/ZnO/InSb CQD/MoO*_x_*/Ag, as illustrated in the device stack in Figure 5a and the cross‐sectional SEM in Figure 5b. The dark and light I–V characteristics of devices are compared in Figure 5c,d. Devices with ME‐exchanged CQDs (denoted as ME devices) showed a rectification ratio of 230, representing a 65% improvement compared to that of MTG devices. The dark current of ME devices at 1 V reverse bias was 0.045 mA cm^−2^, which is 85% lower than that of MTG. The dark current and photocurrent of Na_2_S‐exchanged CQD devices were 1–2 orders of magnitude lower, both on the forward and reverse sides, suggesting poor conductivity of these CQDs.

ME devices achieved an EQE of 28% at 1450 nm at 1 V bias (Figure 5e), slightly higher than the 25% EQE of halide‐passivated InSb CQD photodetectors reported at 1400 nm.^[^ ^10^ ^]^ The EQEs of MTG and Na_2_S devices were 9.5% and 0.6% at their excitonic peaks, respectively. The higher EQE of ME devices compared to those of controls is attributed to the improved carrier mobility and reduced trap density of ME‐passivated InSb CQDs. Device EQE statistics are provided in Table S6.

We further investigated the photodetection characteristics of the ME‐InSb CQD photodetectors. Transient photocurrent responses of devices with 0.1 cm^2^ pixel size showed a fall time of 2.5 µs under 1 V bias (Figure 5f). The responsivity of ME‐InSb CQD photodetectors was calculated based on the EQE spectra, reaching 0.33 A W^−1^ at 1450 nm. From the responsivity and the measured noise current of 9.0 × 10^−12^ A·Hz^−0.5^ at 10 kHz, the specific detectivity was determined to be 1.2 × 10^10^ Jones at 1450 nm (Figure S15).

We note that a number of prior reports assess the operating stability of CQD photodetectors in the absence of applied bias as these CQD devices tend to degrade more quickly in the electric field.^[^ ^35^, ^36^ ^]^ Today, though, the best InSb CQD devices rely on applied bias during operation, and indeed biasing is widespread in applications such as telecommunications, medical imaging, and light detection and ranging (LIDAR), in which fast response, high sensitivity, and low noise are required. To emulate the practical scenarios, we assessed the operating stability of ME‐InSb CQD photodetectors by monitoring their photocurrent under continuous 1450 nm light illumination and reverse bias (Figure 5g). At a reverse bias of 0.5 V, these photodetectors retained 95% of their initial performance after 300 h of continuous operation, representing a 10‐fold improvement in operating lifetime compared to reported halide‐passivated CQD photodetectors (Figure 5h and Table S7). At a higher reverse bias of 1.0 V, the ME‐InSb devices still exhibited stability, maintaining 85% of their initial photocurrent after 100 h of continuous illumination (Figure S16), marking the longest demonstrated operating lifetime among CQD photodetectors under such demanding conditions.

We also compared the thermal stability of thiol‐passivated InSb devices and halide‐passivated InSb devices (Figure S17). After being heated at 50°C for 20 h, the ME‐InSb devices retained 98% of the initial photocurrent, while the halide‐InSb devices dropped to 66%. We posit that the origin of performance loss in halide‐passivated devices is related to heat‐induced halide ion migration. The fact that ME‐InSb devices demonstrate exceptional stability under these demanding conditions (i.e., the presence of electric field, illumination, and mild heat) is significant, showcasing the robustness of thiol‐based surface passivation and the reliability of InSb CQD photodetectors for practical applications.

## Conclusion

In summary, we report the use of nucleophilic covalent ligands for simultaneous surface reconstruction and passivation of InSb CQDs. The selected short‐chain thiol molecules facilitate fast and efficient removal of the oleate ligands and surface oxides via nucleophilic attack while providing strong covalent bonding with surface In and Sb atoms. This leads to enhanced electronic coupling between CQDs and a 10‐fold reduction in trap densities of thiol‐passivated CQDs than the control. Specifically, ME‐passivated InSb CQD ink has a prolonged shelf lifetime of at least 5 months, without any degradation in optical absorption or structural integrity. The photodetectors fabricated with ME‐InSb CQDs as the active layer achieve 28% EQE at 1450 nm, comparable to state‐of‐the‐art heavy‐metal‐free CQD photodetectors at similar wavelengths. The operating stability of our photodetectors is the highest reported to date, showing 95% performance retention after 300 h of operation. This work presents a new approach to resurfacing III‐V CQDs for synergistic improvement of materials stability and device durability.

## Supporting Information

The authors have cited additional references within the Supporting Information.^[^ ^37^, ^38^, ^39^, ^40^, ^41^, ^42^, ^43^, ^44^, ^45^, ^46^, ^47^, ^48^ ^]^

## Conflict of Interests

The authors declare no conflict of interest.

## Supporting information



Supporting Information

## Data Availability

The data that support the findings of this study are available from the corresponding author upon reasonable request.
