# Supplementary material for: Nucleophilic Covalent Ligands Enable Simultaneous Surface Reconstruction and Passivation of Colloidal InSb Quantum Dots for Stable Short‐Wave Infrared Photodetectors
Source: Angew Chem Int Ed Engl. 2025 May 22;64(28):e202505179. doi: 10.1002/anie.202505179 (PMC12232879; doi:10.1002/anie.202505179)
Supplement: Supplementary file 1 — Supporting Information [file ANIE-64-e202505179-s001.pdf]

Supporting Information

Nucleophilic Covalent Ligands Enable Simultaneous Surface Reconstruction and Passivation of Colloidal InSb Quantum Dots for Stable Short-wave Infrared Photodetectors

*Yangning Zhang, Muhammad Imran, Pan Xia, Yiqing Chen, Ahmet Gulsaran, Yanjiang Liu, Ehsan Nikbin, Benjamin Rehl, Lizhou Fan, Filip Dinic, Da Bin Kim, Lewei Zeng, Mustafa Yavuz, Sjoerd Hoogland, Edward H. Sargent**

Table of Contents:

Experimental Details

Figure S1. ToF-SIMS of halide-passivated InSb CQD photodetectors.

Figure S2. TEM of oleic acid-capped InSb CQDs and corresponding size histogram.

Figure S3. XPS spectra of OA-capped InSb CQDs.

Figure S4. Binding energies for different ligands calculated by DFT.

Figure S5. ^{1}H-NMR spectra of pure Na_2S ligands and Na_2S-treated CQDs.

Figure S6. XPS S2p spectra of ligand-exchanged CQDs.

Figure S7. Materials characterization of K_2S-exchanged InSb CQDs.

Figure S8. XPS spectra of the thiol ligand exchange by-products.

Figure S9. STEM images of ligand-exchanged CQDs.

Figure S10. X-ray diffraction patterns of OA-InSb CQDs and ligand-exchanged InSb CQDs.

Figure S11. TA spectral kinetics of ligand-exchanged CQDs.

Figure S12. SCLC characterization of InSb CQDs passivated with different ligands.

Figure S13. Field effect characterizations of InSb CQD films.

Figure S14. TEM images of ME-passivated CQDs after 150 days of storage.

Figure S15. Responsivity, noise, and detectivity of ME-InSb CQD photodetectors.

Figure S16. Operating stability of ME-passivated InSb CQD photodetectors at 1 V.

Figure S17. Comparison of thermal stability between ME-InSb CQD photodetectors and halide-InSb counterparts.

Table S1. Comparison of optical features for InSb CQDs passivated with different ligands.

Table S2. Fitted parameters for the TA data.

Table S3. Fitted parameters for the TRPL data.

Table S4. Trap-filled limit voltage, trap density, and hole mobility calculated from SCLC.

Table S5. Hole mobility of InSb CQDs calculated from FET.

Table S6. Device EQE statistics.

Table S7. Comparison of efficiency and stability vs. prior reports.

Experimental Details

Preparation of stock solutions

$InCl_3/ZnBr_2$ stock solution was prepared by dissolving 440 mg of $InCl_3$ and 1800 mg of $ZnBr_2$ in a mixture of 30 mL oleylamine and 7.5 mL trioctylamine. $SbCl_3$ stock solution was prepared by dissolving 570 mg of $SbCl_3$ in 10 mL oleylamine. Both stock solutions were heated at 70 ºC overnight.

Synthesis of InSb colloidal quantum dots (CQDs)

InSb CQDs were synthesized following a modified hot injection method.[S1] The entire $InCl_3/ZnBr_2$ stock solution was loaded to a round-bottom flask and degassed on the schlenk line at 50 ºC for 10 min. Under argon, 2.3 mL of $SbCl_3$ stock solution was injected into the flask, followed by the injection of 7.5 mL alane N,N-dimethylethylamine complex solution. 5 min later, the reaction temperature was raised from 50 ºC to 260 ºC at a ramping rate of 5 ºC/min. The reaction mixture was then maintained at 260 ºC for 85 min. Finally, the flask was cooled down to room temperature and brought to a nitrogen-filled glovebox for purification. 35 mL of toluene and 8 mL of oleic acid (OA) were added to the reaction product, and the mixture was centrifuged at 6000 rpm for 5 min to remove insoluble by-products. The supernatant was collected and went through 5 cycles of size-selective precipitation using acetonitrile as an antisolvent. The purified CQDs were dispersed in toluene.

Solution-phase thiol ligand exchange

As-synthesized InSb CQDs were purified again with acetonitrile and ethanol, dried, and redispersed in hexane prior to the ligand exchange. Thiol exchange solutions were prepared by adding 50-100 µl of the 2-mercaptoethanol (ME) or methyl thioglycolate (MTG) to 5 ml of N,N-dimethylformamide (DMF). The thiol ligand exchange was performed in a nitrogen-filled glovebox. About 2 mL of CQD hexane solution (15-30 mg/mL) was mixed with 2 mL of the exchange solution. The mixture was vortexed for 2-3 min until the CQDs completely transferred from the hexane phase to the DMF phase. The top hexane phase was removed, followed by 3-4 additional washes with hexane. After ligand exchange, CQDs were precipitated via the addition of toluene and separated by centrifugation. The CQDs were then dried and redispersed in a mixture of DMF and acetonitrile.

Solution-phase metal sulfide ligand exchange

As-synthesized InSb CQDs were purified again with acetonitrile and ethanol, dried, and redispersed in toluene prior to the ligand exchange. Na_2S or K_2S exchange solution was prepared by dissolving 20-30 mg of Na_2S or K_2S in 2 ml of formamide (FA). The Na_2S or K_2S

ligand exchange was performed in a nitrogen-filled glovebox. About 2 mL of CQD toluene solution (15-20 mg/mL) was mixed with 1.5 mL of the exchange solution and 0.5 mL of formamide. The mixture was vortexed for 2-3 min. The separation between the non-polar phase and polar phase was very slow, so the mixture was centrifuged at 4000 rpm for 1 min to help separate the two phases. After the CQDs completely transferred from the toluene phase to the formamide phase, the top toluene phase was removed, followed by 3-4 additional washes with toluene. After ligand exchange, CQDs were precipitated via the addition of acetonitrile and separated by centrifugation. The CQDs were then dried and redispersed in a mixture of formamide and butylamine.

Device fabrication

Pre-patterned indium tin oxide (ITO) glass substrates were cleaned with DI water, acetone, and isopropanol respectively, and dried before use. For the electron transporting layer (ETL), the ZnO nanoparticles were synthesized following a published method.[S2] To deposit two layers of ZnO, the filtered ZnO nanoparticles were spin-coated on ITO substrates at 5000 rpm for 30 s in air, and the process was repeated twice to achieve a thickness of 150 nm. For the InSb CQD active layer, 30-40 μL of ligand-exchanged CQD ink with a concentration of 150-200 mg/mL was spin-coated on top of the ZnO layer at 800-1500 rpm inside a nitrogen-filled glovebox. The active layer thickness is in the range of 130-160 nm. The films were then annealed at 80 °C for 20 min to get rid of excess solvents. For the hole transporting layer (HTL), MoO_x was deposited on top of the CQD layer by thermal evaporation with a deposition rate of 0.4 Å/s to reach a thickness of 14 nm. Finally, a 140 nm layer of silver was thermally evaporated on top of HTL as the top electrode. The device active area is 0.1 cm^2. The devices were encapsulated before measurements.

Materials characterization

The optical absorption measurements of InSb CQD solutions were performed with a Perkin-Elmer Lambda 1050 UV/Vis Spectrometer.

Steady-state photoluminescence (PL) spectra of InSb CQD solutions were collected using a Horiba Fluorolog system equipped with an NIR photomultiplier tube detector (H10330C-75, Hamamatsu). A monochromatized Xe lamp was used as the excitation source. Time-resolved PL measurements were performed using a time-correlated single photon counting (TCSPC) detector and a pulsed NIR laser diode (820 nm, Horiba).

For transient absorption (TA) measurements, a regeneratively amplified Yb:KGW laser at a 5 kHz repetition rate (Light Conversion, Pharos) was used to generate femtosecond laser

pulses. The samples were photoexcited with a 130 μW 560 nm pump pulse. The time delay between pump and probe was adjusted by changing the path length of the probe (time resolution ~350 fs). The probe pulse was then collected by a CCD after dispersion by a grating spectrograph (Ultrafast).

High-resolution transmission electron microscopy (TEM) and scanning transmission electron microscopy (STEM) images of the CQD samples were acquired with a Hitachi HF-3300 TEM. Cross-sectional scanning electron microscopy (SEM) images of InSb CQD photodetectors were taken using a Hitachi SU3500 SEM.

Fourier-transform infrared spectroscopy (FTIR) measurements were performed in the attenuated total reflectance (ATR) mode using an FTIR spectrometer (Thermo Scientific iS50).

X-ray Photoelectron Spectroscopy (XPS) was measured using a Thermofisher Scientific Escalab 250Xi system equipped with an Al Kα X-ray source (1486.6 eV). XPS samples were prepared by depositing InSb CQDs on ITO substrates.

^{1}H Nuclear Magnetic Resonance (NMR) spectra were recorded with an Agilent DD2 600 MHz NMR Spectrometer. OA-capped InSb CQDs and pure OA ligands were dispersed in d-benzene for NMR measurements. ME-exchanged InSb CQDs and pure ME ligands were dispersed in d-DMF. Na_2S-treated CQDs and pure Na_2S ligands were dispersed in D_2O.

Time-of-flight secondary-ion-mass spectrometry (ToF-SIMS) measurements of InSb CQD photodetectors were performed with a ToF-SIMS spectrometer (ToF-SIMS V system, ION-TOF). Cs^+ ions with 1000 eV ion energy were used for depth profiling.

Grazing-incidence small-angle and wide-angle X-ray scattering (GISAXS/GIWAXS) patterns of InSb CQD films were collected at the BXDS beamline at the Canadian Light Source, with an X-ray energy of 9.8 keV. The scattering patterns were obtained at an incident angle of 0.25 degrees with respect to the sample plane.

X-ray diffraction (XRD) measurements were performed using a Rigaku MiniFlex 600 powder X-ray diffractometer with monochromatized Cu Kα radiation.

Space-charge limited current (SCLC) measurements were conducted by measuring the dark I-V characteristics of hole-only devices with ITO/EDT-PbS CQD/InSb CQD/MoOx/Ag structure. EDT-PbS CQDs are prepared by a layer-by-layer ligand exchange process using 1,2-ethanedithiol ligands.[S3]

Bottom-gate top contact FET substrates were fabricated with UV lithography method to pattern the electrodes on thermal SiO_2 (291 nm)/Si(p^{++}) wafers. The channel was patterned as interdigitated electrodes (IDE) structure to increase the width-to-length (W/L) ratio by having multiple fingers. The dimensions of channel width W=100 μm, channel length L=2 μm, and

625 fingers (i.e. 1249 channels) resulted in a high width-to-length ratio of 62450 (calculated by n*W/L). Ligand-exchanged InSb CQDs were deposited on top of FET substrates and annealed at 90 ºC for 1 h. A parameter analyzer was used to measure the transfer curves with a fixed drain-source voltage (V_{DS}= +35 V). The output curves were measured by scanning the I_{DS}-V_{DS} characteristics under different gate voltages (V_{GS}). The source was grounded during the measurements.

Device characterization

Current-voltage (I-V) characteristics of InSb CQD photodetectors were measured using a Keithley 2400 source meter in the dark or under illumination (1450 nm laser, 10 mW/cm^2). The I-V curves were scanned from -1 V to +1 V with a step size of 0.02 V.

External quantum efficiency (EQE) measurements were performed using a Quantum Efficiency Measurement System (QE-R, Enlitech). EQE spectra were taken in the wavelength range of 500-1800 nm with a step size of 10 nm by subjecting the devices to chopped (210 Hz) monochromatic illumination.

The temporal response of the photodetectors was collected using a 1-GHz oscilloscope (DSO8104A Infinium, Agilent). Pixels were illuminated with a 1310 nm diode laser (ML725B8F, Thorlabs) modulated at a frequency of 10 kHz using a function generator (33220A, Agilent). An illumination power of 10 mW/cm^2 was used. The photodetector signal was pre-amplified (10^3 A V^{-1}) using a pre-amplifier (Femto DHPCA-100). A bias voltage of 1.0 V was applied through the pre-amplifier.

Noise measurements were performed using a low-noise preamplifier (SR570, Stanford Research Systems) connected with a lock-in amplifier (SR830, Stanford Research Systems) under a reverse bias of 1.0 V across the frequency range of 50 Hz-100 kHz. Each datapoint was obtained using a measurement bandwidth of 1 Hz. The noise current was calculated from the measured noise voltage values with a sensitivity of 1×10^{-5} A V^{-1}. The detectivity was determined using the noise current, responsivity, and an active area of 0.1 cm^2.[S4]

The operating stability of encapsulated photodetectors was assessed by monitoring the light current as a function of time under monochromatic illumination with a 1450 nm laser (10 mW/cm^2). A reverse bias voltage of 0.5 V or 1.0 V was applied throughout the measurements.

Density functional theory (DFT) simulations

DFT simulations were performed using the projector-augmented wave method[S5] as implemented in the Vienna Ab initio Simulation Package (VASP).[S6] The Perdew-Burke-Ernzerhof (PBE) functional of generalized gradient approximation (GGA) was applied to

describe the exchange-correlation interactions.[S7] A plane-wave kinetic energy cutoff of 450 eV was used throughout the calculations. To incorporate van der Waals forces, the DFT-D3 (zero) method was employed.[S8] Structural relaxations of the slab models were performed until the residual Hellmann-Feynman forces on each atom were smaller than 0.02 eV· $Å^{-1}$, and the total energy converged to within 10^{-5} eV/atom. The (111) surface of InSb was chosen to model different passivation schemes, using a six-layer (2x2) surface model. For ligand adsorption, the In-terminated surface was considered, while the dangling bonds on the Sb-terminated side were passivated using pseudo-hydrogen atoms with a charge of 0.75 electrons. A vacuum layer greater than 15 Å was introduced to eliminate any spurious interactions between periodic images of the slab.

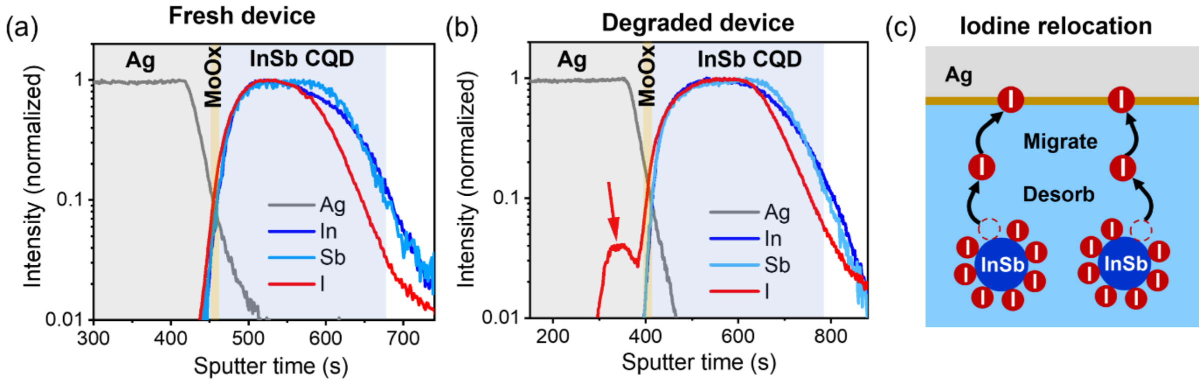

Figure S1. Time-of-Flight Secondary Ion Mass Spectrometry (ToF-SIMS) studies of (a) fresh and (b) degraded halide-passivated InSb CQD photodetectors. The device configuration is ITO/ZnO/halide-InSb CQD/MoOx/Ag, in which InSb CQDs were treated with malonic acid and tetrabutylammonium iodide sequentially according to our previous study.[S9] In (a), the fresh device showed the localization of iodine ions within the InSb layer. In (b), the iodine signals in the Ag layer suggest iodine migration from the InSb layer to the Ag electrode after device operation. (c) Schematic illustration of iodine ion desorption and relocation. The reaction between halides and Ag reduces conductivity, leading to device performance degradation.

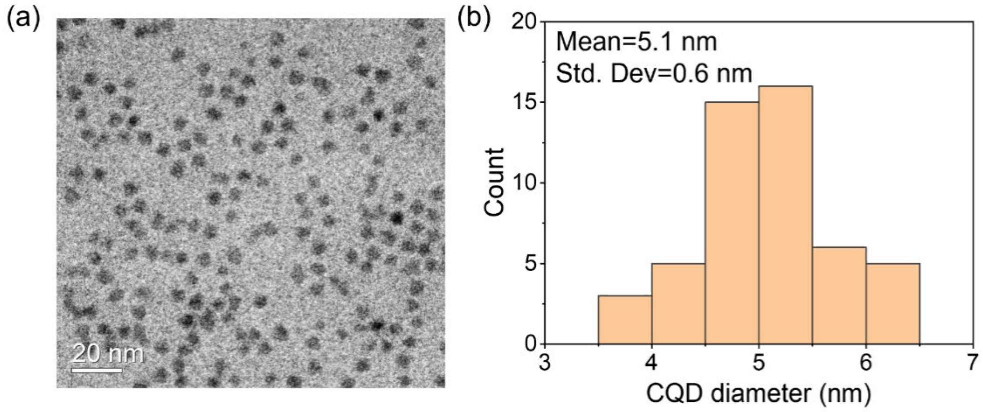

Figure S2. (a) Transmission electron microscopy (TEM) image of oleic acid (OA)-capped InSb CQDs and (b) histogram of CQD size distribution. The average diameter of InSb CQDs is 5.1 nm with a standard deviation of 0.6 nm.

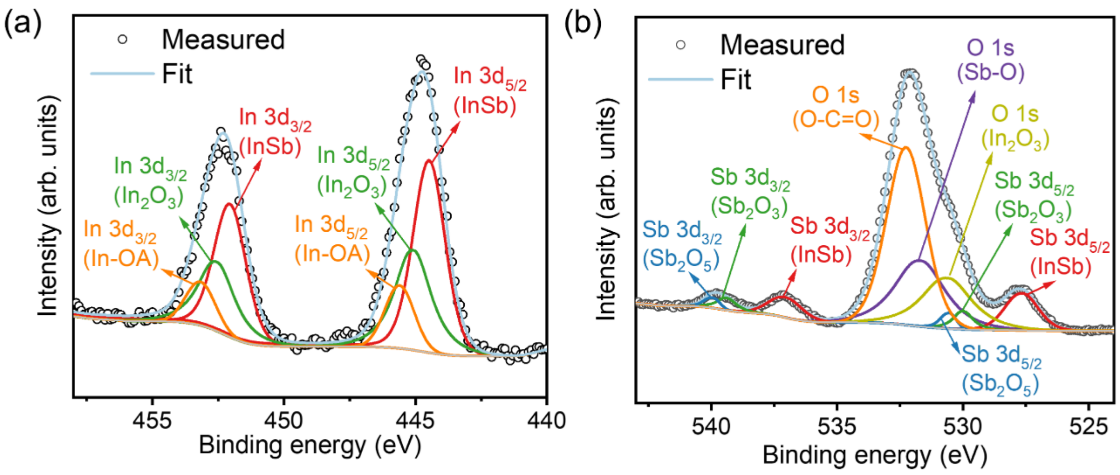

Figure S3. XPS spectra of OA-capped InSb CQDs: (a) In 3d, and (b) Sb 3d. Each of the In $3d_{3/2}$ and In $3d_{5/2}$ peaks are deconvoluted into InSb, In_2O_3, and In-oleate components. The Sb 3d spectrum shows the coexistence of Sb_2O_x (x=3, 5) and InSb. The O 1s signals are deconvoluted into metal oxides and oleate ligands.

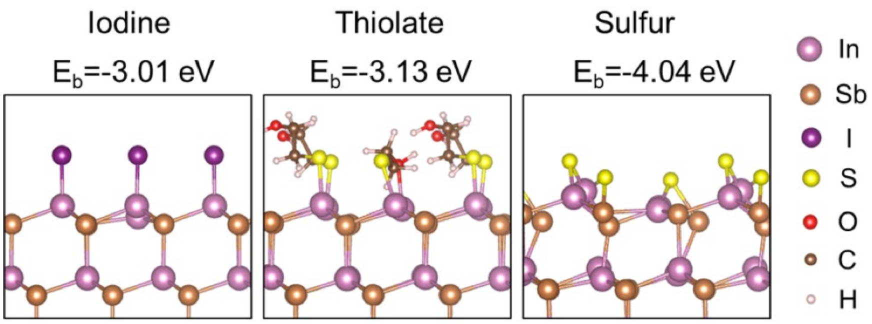

Figure S4. Binding energies (E_b) for different ligands (iodine, thiolate, sulfur) to InSb (111) surface calculated by DFT. In view of the multiple binding sites, the S^{2-} anions bind more strongly than I^- anions to the InSb surface. This indicates that S^{2-} anions may be less likely to detach from the CQD surfaces. The binding energy of thiolate to InSb is 0.12 eV higher than that of I^-, and the size of thiolate anion is much larger. The I^- anions, being small and not sterically hindered, can easily desorb from the CQDs and migrate across the CQD film. In contrast, the steric hindrance of the organic backbone is expected to suppress the movement of thiolate anions. Therefore, we posit that using sulfur-based ligands to passivate InSb CQDs could suppress ligand desorption and migration, enhancing the stability of InSb CQD photodetectors.

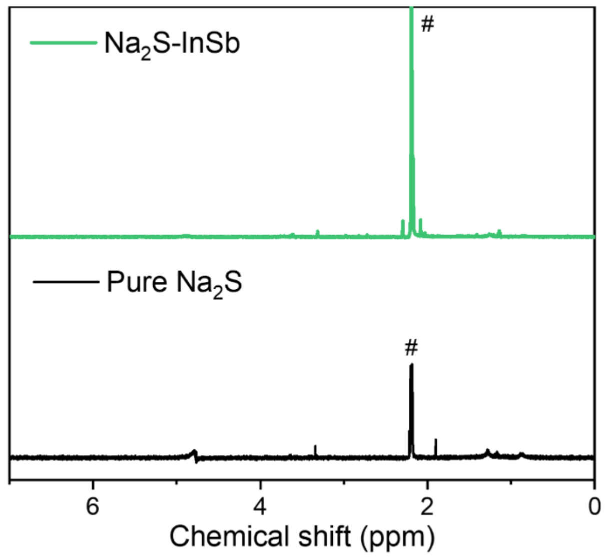

Figure S5. ^{1}H-NMR spectra of pure Na_2S ligands and Na_2S-treated CQDs. No characteristic proton signals are observed for inorganic Na_2S ligands. In Na_2S-treated CQDs, the absence of signals in the 5.2–5.6 ppm range (corresponding to the C=C protons of oleate) indicates substantial removal of oleate ligands. The proton signal from residual acetone solvent is marked with #.

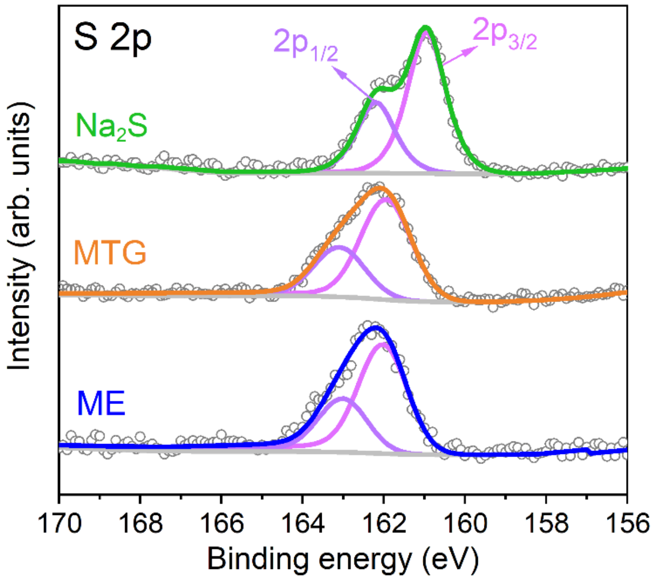

Figure S6. XPS S2p spectra of ME-, MTG-, and Na_2S-exchanged CQDs. The S 2p core level is deconvoluted into S $2p_{1/2}$ and S $2p_{3/2}$ components. For ME- and MTG-treated CQDs, the S $2p_{3/2}$ component at 162 eV is characteristic of thiolate bound to metal. For Na_2S-exchanged CQDs, the spin–orbit splitting is more pronounced, and the S $2p_{3/2}$ component at 161 eV is expected for metal sulfides.

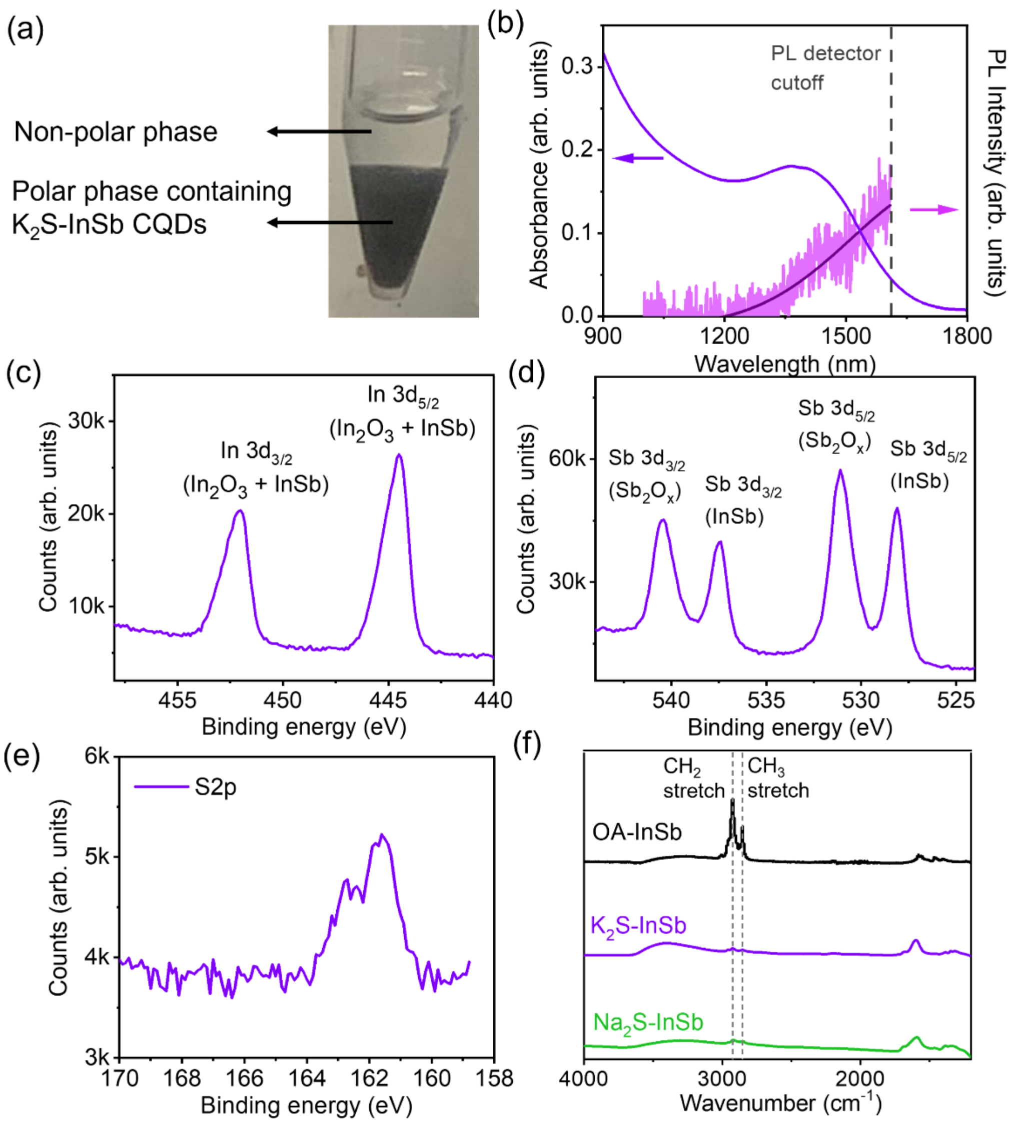

Figure S7. (a) Photograph of K_2S ligand exchange for InSb CQDs. The CQDs have completely transferred from the non-polar phase to the bottom polar phase. (b) Absorbance and PL spectra of K_2S exchanged CQDs. The PL emission was extremely weak, with a Stokes shift of more than 200 nm. XPS spectra of K_2S exchanged CQDs: (d) In 3d spectra, (d) Sb 3d spectra, and (e) S 2p spectra. The CQDs have a significant amount of remaining surface metal (In/Sb) oxides. (f) FTIR spectra of K_2S exchanged CQDs in comparison with OA-capped and Na_2S exchanged CQDs. The drastic decrease of CH_2 and CH_3 signals in the exchanged CQDs suggests that native OA ligands have been mostly removed. The broad peak at 3320 cm^{-1} and the sharp peak at 1595 cm^{-1} arise from the residual formamide solvent.

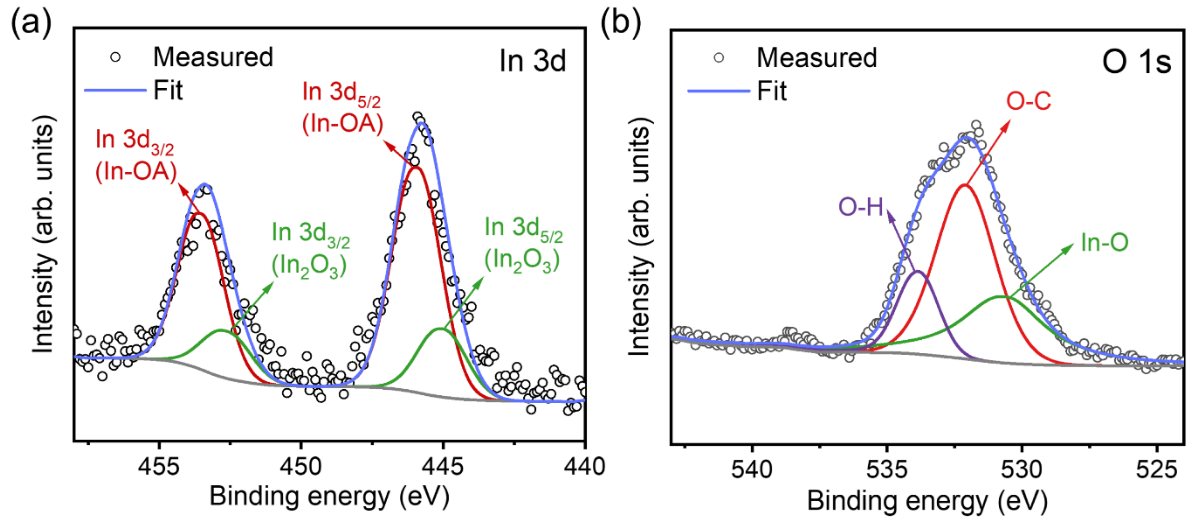

Figure S8. XPS spectra of the thiol ligand exchange by-products (i.e. dried supernatant collected during the thiol ligand exchange): (a) In 3d and (b) O 1s. The In peaks correspond to In-oleate and In_2O_3. The O 1s peak consists of multiple components, including the In-O bond from In_2O_3, the O-C bond from oleic acid or In-oleate, and the O-H bond from water. The absence of Sb 3d peaks at 527 and 537 eV indicates that the amount of Sb is negligible in the supernatant.

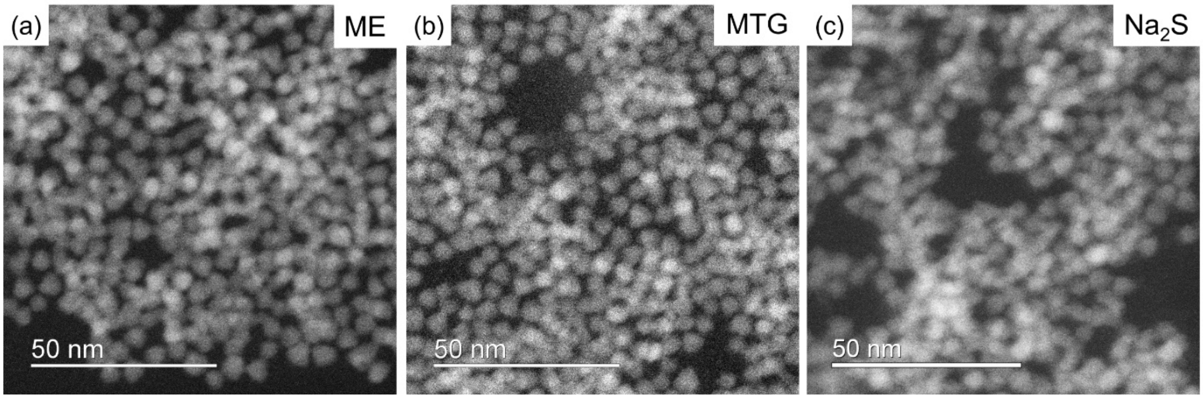

Figure S9. Scanning transmission electron microscopy (STEM) images of InSb CQDs passivated with different ligands: (a) ME, (b) MTG, or (c) Na_2S. The CQDs exhibit the same size and shape as the OA-capped ones. The average interparticle distances have decreased to 5.5 nm for ME-InSb CQDs, 5.8 nm for MTG-InSb CQDs, and 6.1 nm for Na_2S-InSb CQDs.

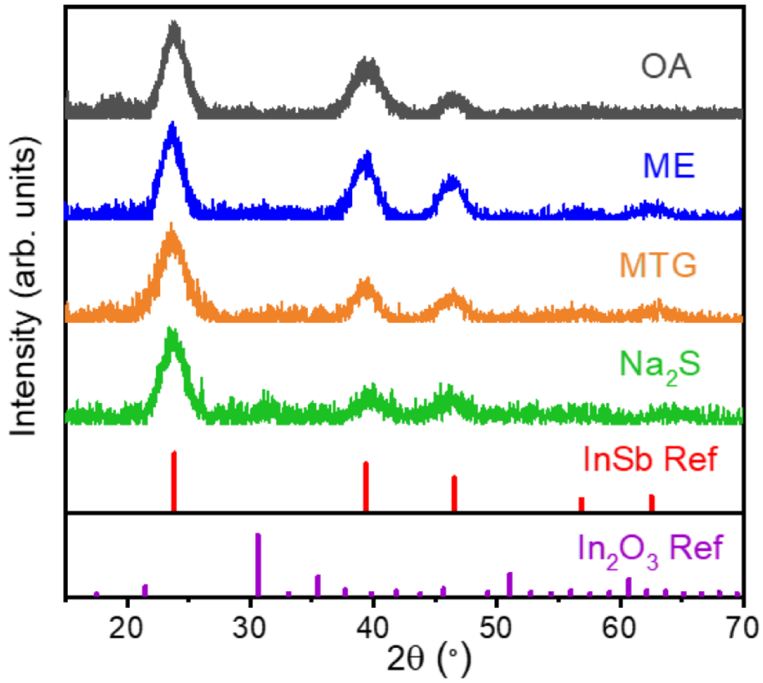

Figure S10. X-ray diffraction patterns of OA-InSb CQDs and InSb CQDs exchanged with different ligands (ME, MTG, or Na_2S). The diffraction peaks match with the zinc blende crystal structure of InSb (ICCD PDF# 06-0208). No obvious In_2O_3 (ICCD PDF# 06-0416) peaks were observed.

Table S1. Comparison of absorbance peak center, PL peak center, and Stokes shift for InSb CQDs passivated with different ligands.

CQD sample	Absorbance peak center (nm)	PL peak center (nm)	Stokes shift (nm)
OA-InSb	1375	1500	125
ME-InSb	1395	1536	141
MTG-InSb	1400	1543	143
Na_2S -InSb	1435	>1600 nm	>165

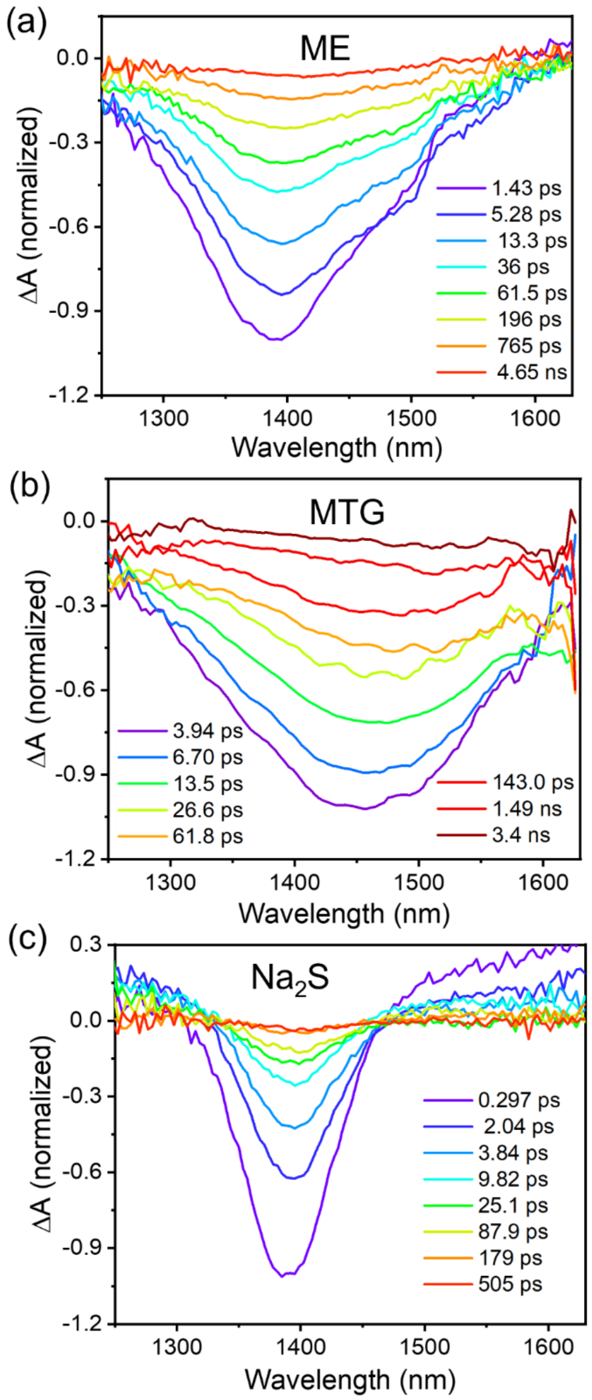

Figure S11. Transient absorption (TA) spectral kinetics of ME-, MTG-, and Na_2S-exchanged CQDs. For Na_2S-exchanged CQDs, the exciton bleach appears narrower than the steady-state absorption spectra in Figure 3a. This suggests that a sub-picosecond trapping process has happened beyond the detection limit due to the high density of surface traps in these CQDs.

Table S2. Fitted parameters for the TA data.

Parameter	ME-InSb	MTG-InSb	Na_2S -InSb
y_0	0.02±0.02	0.04±0.01	0.03±0.00
A_1	0.41±0.01	0.36±0.03	0.72±0.04
A_2	0.36±0.01	0.37±0.03	0.14±0.03
A_3	0.22±0.01	0.22±0.02	0.14±0.03
τ_1 (ps)	7.6±0.4	7.5±1.1	2.3±0.1
τ_2 (ps)	66.0±3.3	78.7±12.5	14.2±7.5
τ_3 (ps)	1555.0±15.0	1461.9±298.9	100.0±24.7
Average lifetime (ps)	1447.6	1338.7	80.9

Note: In the TA analysis, the ground-state bleaching recovery dynamics are fitted with three components. The ultrafast decay (<10 ps) is attributed to Auger recombination or biexciton decay, consistent with previous reports.[S10] The intermediate (10-100 ps) and long-lived (1-2 ns) components are currently ascribed to trapping-related processes based on prior studies.[S10, S11] These two components are 5-15 times longer in thiol-treated CQDs than in Na_2S-treated CQDs, suggesting more effective passivation of surface traps by thiolate ligands.

Table S3. Fitted parameters for the transient photoluminescence (TRPL) data.

Parameter	ME-InSb	MTG-InSb
y_0	0.008±0.001	0.009±0.001
A_1	0.627±0.008	0.589±0.010
A_2	0.235±0.006	0.300±0.008
A_3	0.150±0.005	0.133±0.005
τ_1 (ns)	1.27±0.03	1.33±0.03
τ_2 (ns)	8.81±0.45	6.75±0.28
τ_3 (ns)	54.70±2.54	35.74±1.15
Average lifetime (ns)	42.3	24.4

Note: TRPL measurements of thiol-treated CQDs reveal two fast decay components (1-2 ns and 6-9 ns) that are attributed to non-radiative recombination processes, and a slower component (30~60 ns) corresponding to radiative recombination.[S9, S10]

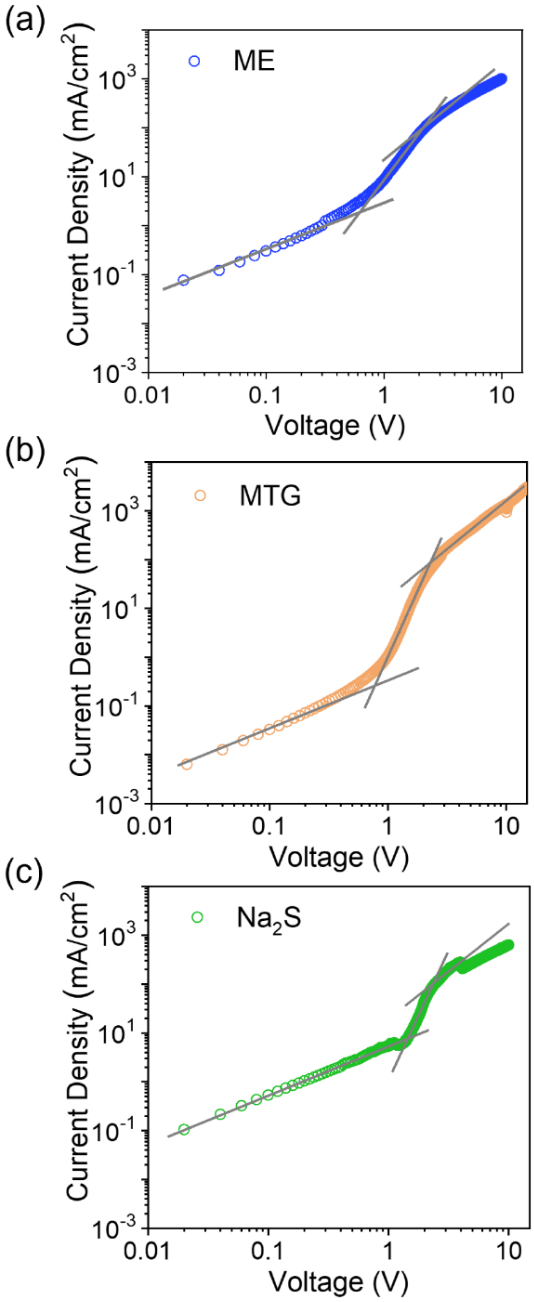

Figure S12. Space-charge limited current (SCLC) of hole-only devices based on ligand-exchanged CQDs. The grey lines represent the fits of three regimes: the Ohmic regime with a slope of 1, the trap-filling regime with a slope>2, and the SCLC regime with a slope of 2.

Table S4. Trap-filled limit voltage (V_{TFL}), trap density, and hole mobility calculated from SCLC measurements.

	V_{TFL} (V)	Trap density (cm^{-3})	Hole mobility ($cm^2\ V^{-1}\ s^{-1}$)
ME-InSb	0.63	1.86×10^{16}	1.15×10^{-4}
MTG-InSb	0.77	2.27×10^{16}	9.13×10^{-5}
Na_2S-InSb	1.44	1.49×10^{17}	1.71×10^{-5}

Note:

The trap-filled limit voltage V_{TFL} was determined from the intersection of the Ohmic regime and trap-filling regime. The trap density n_t was calculated using Equation S1.

$$n_t = \frac{V_{TFL}\varepsilon\varepsilon_0}{eL^2} \quad \text{(S1)}$$

where ε is the relative dielectric constant (we used ε=6 reported for InAs CQDs[S12] as an approximate for InSb CQDs), ε_0 is the vacuum permittivity, and L is the thickness of the CQD layer (L=150 nm for ME-InSb and MTG-InSb; L=80 nm for Na_2S-InSb).

The hole mobility μ was calculated from the SCLC regime based on Equation S2.

$$J = \frac{9}{8}\varepsilon\varepsilon_0\mu\frac{V^2}{L^3} \quad \text{(S2)}$$

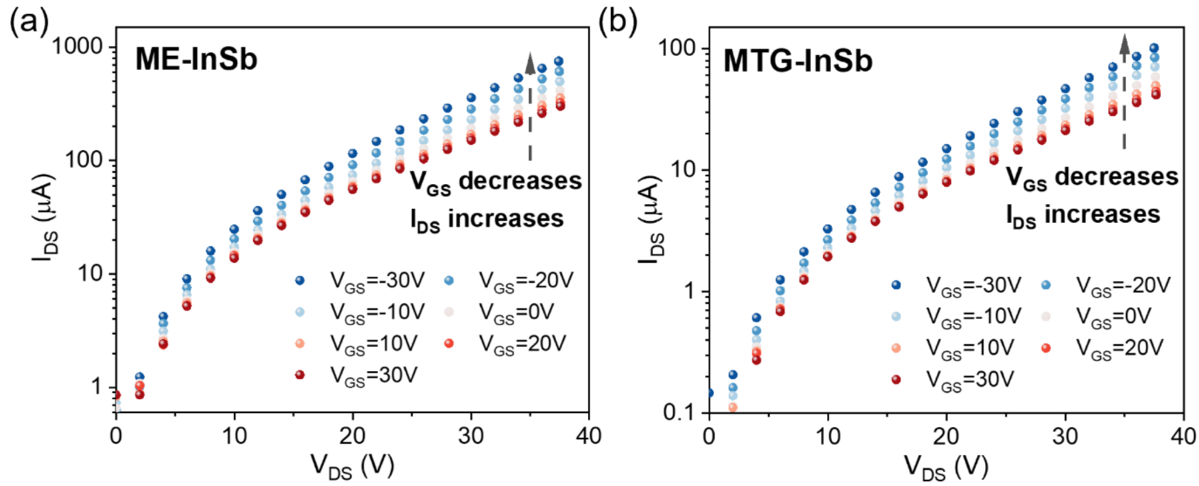

Figure S13. Field effect characterizations of InSb CQD films. Output curves of (a) ME-InSb and (b) MTG-InSb CQDs. The applied gate-source voltages (V_{GS}) are indicated in the legends. For both samples, the drain-source current (I_{DS}) increases with decreasing V_{GS}, suggesting the p-type behavior of the CQDs.

Table S5. Hole mobility of ME-InSb and MTG-InSb CQDs calculated from FET measurements.

	Hole mobility (cm^2 V^{-1} s^{-1})
ME-InSb	7.8×10^{-4}
MTG-InSb	1.1×10^{-4}

Note:

The CQD-based transistors were operated at the linear regime where the drain current is approximately proportional to the gate voltage using Equation S3:

$$I_{DS,lin} = \mu C_i \frac{W}{L}\left((V_{GS} - V_{th})V_{DS} - \frac{V_{DS}^2}{2}\right) \quad \text{(S3)}$$

where $C_i = \frac{\epsilon_r \epsilon_0}{t}$ represents the capacitance per unit area and equivalent to 11.9 nF·cm^{-2} by assuming the relative permittivity of SiO_2 is ϵ_{r,SiO_2} =3.9. V_{th} is the threshold voltage.

The carrier mobility was calculated using the slope of the linear regime of transfer curves, $m_{lin} \equiv \frac{dI_{DS}}{dV_{GS}}$ (also called transconductance), as shown in Equation S4.

$$\mu = m_{lin} \frac{L}{W} \frac{1}{V_{DS}} \frac{1}{C_i} \quad \text{(S4)}$$

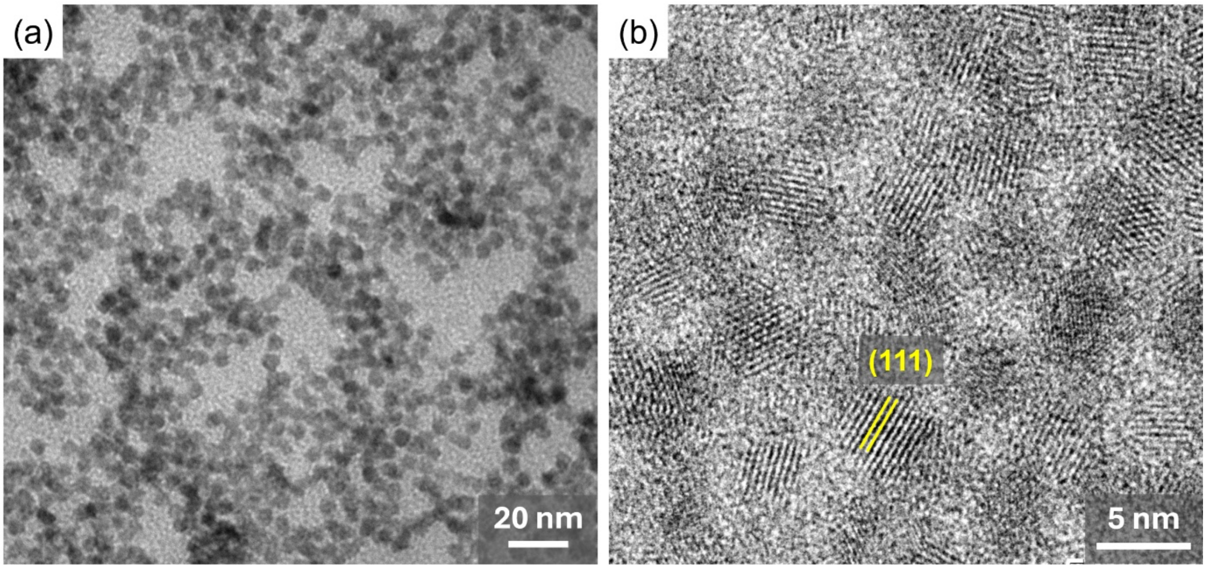

Figure S14. Transmission electron microscopy (TEM) images of ME-passivated InSb CQDs after 150 days of storage. The size, shape, and crystal structure of these CQDs remained the same as those of fresh CQDs.

Table S6. Average values and standard deviation of EQE of InSb CQD photodetectors with different surface passivation (sample size=5).

	ME-InSb devices	MTG-InSb devices	Na_2S-InSb devices
EQE (%) at 1V	27.6±4.6	9.5±1.6	0.6±0.3

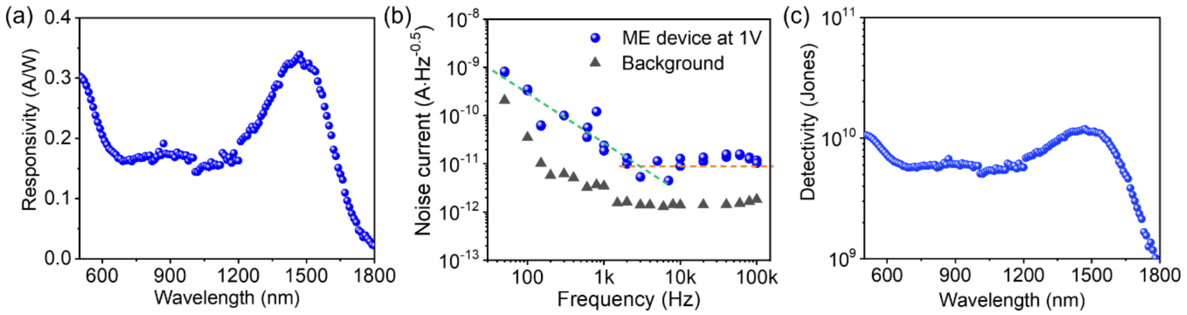

Figure S15. (a) Responsivity, (b) noise spectra, and (c) detectivity of ME-InSb CQD photodetectors. The responsivity reaches 0.33 A/W at 1450 nm. The noise current of 9.0×10^{-12} $A\cdot Hz^{-0.5}$ at a frequency of 10 kHz was used for detectivity calculation, as this frequency lies within the device's operational range (~10 kHz). The calculated specific detectivity is 1.2×10^{10} Jones at 1450 nm.

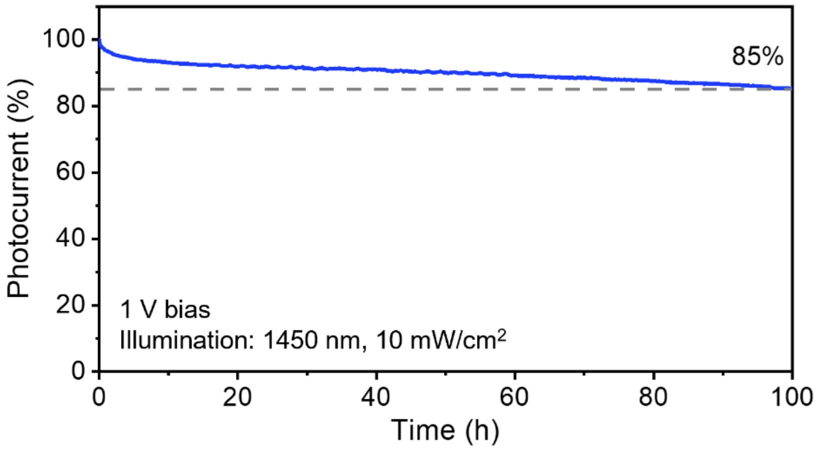

Figure S16. Operating stability of ME-passivated InSb CQD photodetectors at 1 V reverse bias. The devices retained 85% of initial photocurrent after 100 h of continuous biased and illuminated operation.

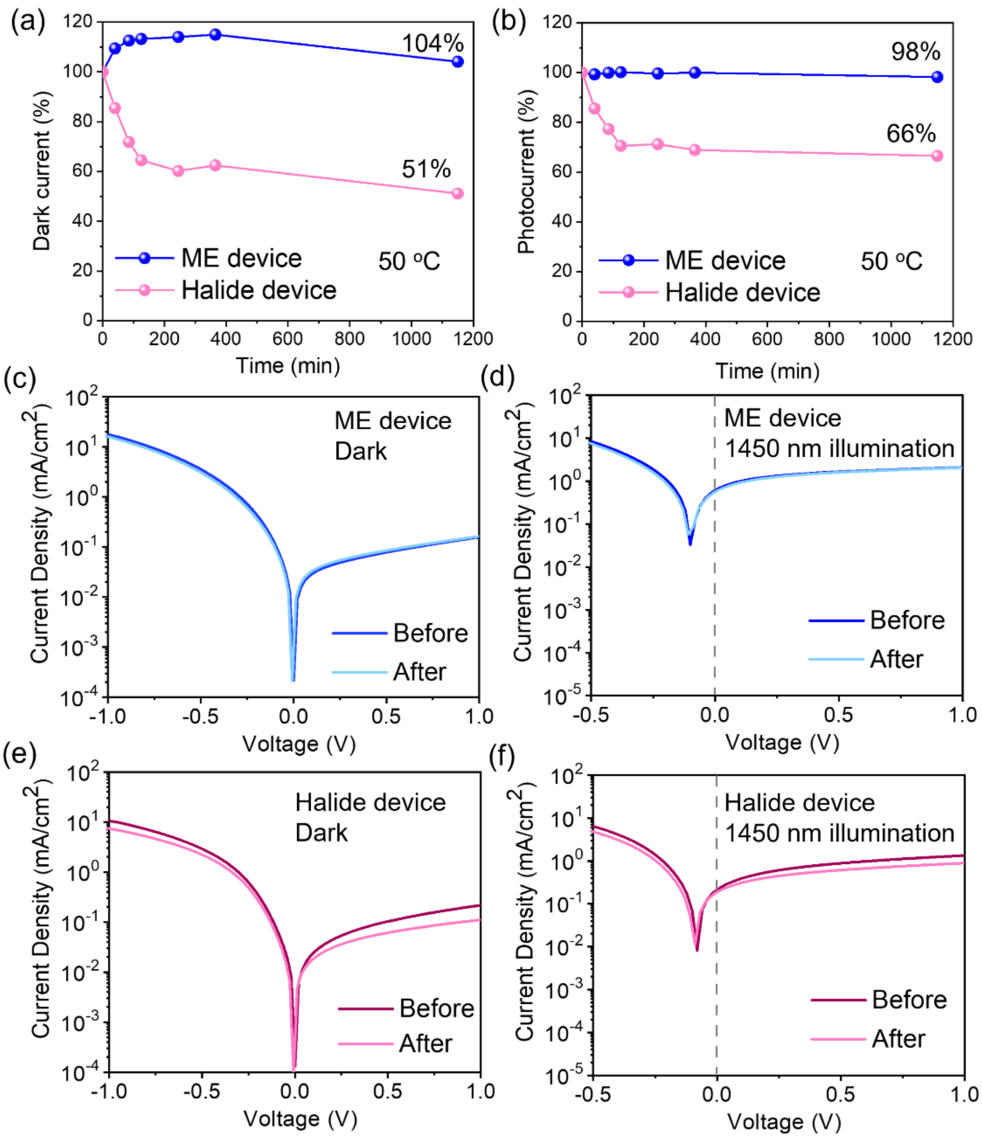

Figure S17. Device thermal stability. Comparison of (a) dark current and (b) photocurrent stability of a ME-InSb CQD photodetector and a halide-InSb CQD photodetector. The halide-InSb photodetector was fabricated following our previously reported procedures.[S9] Both types of devices were heated continuously at 50 °C for 1150 min. (c) Dark and (d) light I-V curves of ME-InSb device before and after the thermal stability test. (e) Dark and (d) light I-V curves of halide-InSb device before and after the thermal stability test.

Note:

The initial increase in dark current observed in the ME device is attributed to thermally activated carrier transport, including enhanced hopping and thermionic emission.[S13] The subsequent decrease in both dark current and photocurrent around 400 min likely results from mild ligand desorption and interfacial degradation under prolonged heating. However, this

decrease is relatively minor compared to the control device, suggesting more robust surface passivation provided by ME ligands.

In contrast, the halide-based control device shows a continuous and more drastic decrease in both dark current and photocurrent. At elevated temperatures, halide ions tend to desorb and migrate, which compromises surface passivation and increases nonradiative recombination.[S14] These effects impede carrier transport and extraction, leading to a significant reduction of currents over time.

Table S7. Comparison of device performance and operating stability vs. prior studies of CQD SWIR photodetectors.

Active layer CQDs	Ligands	Wave-length (nm)	EQE (%)	Dark current (mA/cm^2)	Operating stability			Ref
					Photocurrent retention and time	Illumination condition	Bias (V)	
**InSb**	**Thiol**	**1450**	**28**	**0.05**	**95%, 300 h**	**1450 nm 10 mW/cm^2**	**0.5**	**This work**
InSb	Acid +halide	1400	25	0.001	77%, 10 h	1450 nm 10 mW/cm^2	0.5	[S9]
InSb	Acid +halide	1380	33	0.014	90%, 19 h	1450 nm 10 mW/cm^2	0.5	[S15]
InAs	Halide	1100	31	0.0004	88%, 3 h	0.02 sun	0	[S16]
In(As/P)	Thiol +amine	1400	0.7	0.02	N/A	N/A	N/A	[S17]
InAs /ZnSe	Dithiol	1450	15	0.02	110%, 3h	520 nm 1 mW/cm^2	0	[S18]
PbS	Halide	1450	66	0.001	77%, 16 h	825 nm power N/A	0.3	[S19]
PbS	Halide	1550	80	0.01	70%, 12 h	940 nm 750mW/cm^2	0	[S20]

References:

[S1] Muhammad, D. Choi, D. H. Parmar, B. Rehl, Y. Zhang, O. Atan, G. Kim, P. Xia, J. M. Pina, M. Li, Y. Liu, O. Voznyy, S. Hoogland, E. H. Sargent, *Adv. Mater.* **2023**, *35*, 2306147.

[S2] G. Shi, Y. Wang, Z. Liu, L. Han, J. Liu, Y. Wang, K. Lu, S. Chen, X. Ling, Y. Li, S. Cheng, W. Ma, *Adv. Energy Mater.* **2017**, *7*, 1602667.

[S3] J. Z. Fan, N. T. Andersen, M. Biondi, P. Todorović, B. Sun, O. Ouellette, J. Abed, L. K. Sagar, M. J. Choi, S. Hoogland, F. P. G. de Arquer, E. H. Sargent, *Adv. Mater.* **2019**, *31*, 1904304.

[S4] M. Biondi, M. J. Choi, Z. Wang, M. Wei, S. Lee, H. Choubisa, L. K. Sagar, B. Sun, S. W. Baek, B. Chen, P. Todorović, A. M. Najarian, A. Sedighian Rasouli, D. H. Nam, M. Vafaie, Y. C. Li, K. Bertens, S. Hoogland, O. Voznyy, F. P. García de Arquer, E. H. Sargent, *Adv. Mater.* **2021**, *33*, 2101056.

[S5] P. E. Blöchl, *Phys. Rev. B* **1994**, *50*, 17953.

[S6] a) G. Kresse, J. Furthmüller, *Phys. Rev. B* **1996**, *54*, 11169; b) G. Kresse, D. Joubert, *Phys. Rev. B* **1999**, *59*, 1758.

[S7] a) J. P. Perdew, K. Burke, M. Ernzerhof, *Phys. Rev. Lett.* **1996**, *77*, 3865; b) J. P. Perdew, J. A. Chevary, S. H. Vosko, K. A. Jackson, M. R. Pederson, D. J. Singh, C. Fiolhais, *Phys. Rev. B* **1992**, *46*, 6671.

[S8] S. Grimme, J. Antony, S. Ehrlich, H. Krieg, *J. Chem. Phys.* **2010**, *132*, 154104.

[S9] Y. Zhang, P. Xia, B. Rehl, D. H. Parmar, D. Choi, M. Imran, Y. Chen, Y. Liu, M. Vafaie, C. Li, O. Atan, J. M. Pina, W. Paritmongkol, L. Levina, O. Voznyy, S. Hoogland, E. H. Sargent, *Angew. Chemie Int. Ed.* **2024**, *63*, e202316733.

[S10] A. Y. Chang, W. Liu, D. V Talapin, R. D. Schaller, *ACS Nano* **2014**, *8*, 8513–8519.

[S11] Muhammad, D. Choi, D. H. Parmar, B. Rehl, Y. Zhang, O. Atan, G. Kim, P. Xia, J. M. Pina, M. Li, Y. Liu, O. Voznyy, S. Hoogland, E. H. Sargent, *Adv. Mater.* **2023**, *35*, 2306147.

[S12] B. Sun, A. M. Najarian, L. K. Sagar, M. Biondi, M. J. Choi, X. Li, L. Levina, S. W. Baek, C. Zheng, S. Lee, A. R. Kirmani, R. Sabatini, J. Abed, M. Liu, M. Vafaie, P. Li, L. J. Richter, O. Voznyy, M. Chekini, Z. H. Lu, F. P. García de Arquer, E. H. Sargent, *Adv. Mater.* **2022**, *34*, 2203039.

[S13] Y. Wang, H. Wu, C. Rodà, L. Peng, N. Taghipour, M. Dosil, G. Konstantatos, *Adv. Mater.* **2025**, 2500977.

[S14] L. Chen, J. Liu, P. Liu, S. Lu, Y. Yang, X. Liang, L. Zhang, J. Hu, J. Yang, Y. Liu, W. Ma, X. Zhao, X. Lan, J. Zhang, L. Gao, J. Tang, *ACS Photonics* **2023**, *10*, 2374–2381.

[S15] M. Imran, D. B. Kim, P. Xia, F. Y. Villanueva, B. Rehl, J. M. Pina, Y. Liu, Y. Zhang, O. Voznyy, E. Kumacheva, S. Hoogland, E. H. Sargent, *Adv. Mater.* **2025**, *37*, 2420273.

[S16] M. Vafaie, A. Morteza Najarian, J. Xu, L. J. Richter, R. Li, Y. Zhang, M. Imran, P. Xia, H. W. Ban, L. Levina, A. Singh, J. Meitzner, A. G. Pattantyus-Abraham, F. P. García de Arquer, E. H. Sargent, *Proc. Natl. Acad. Sci.* **2023**, *120*, e2305327120.

[S17] J. Leemans, V. Pejović, E. Georgitzikis, M. Minjauw, A. B. Siddik, Y. H. Deng, Y. Kuang, G. Roelkens, C. Detavernier, I. Lieberman, P. E. Malinowski, D. Cheyns, Z. Hens, *Adv. Sci.* **2022**, *9*, 2200844.

[S18] T. Sheikh, W. J. Mir, A. Alofi, M. Skoroterski, R. Zhou, S. Nematulloev, M. N. Hedhili, M. Ben Hassine, M. S. Khan, K. E. Yorov, B. E. Hasanov, H. Liao, Y. Yang, A. Shamim, M. Abulikemu, O. F. Mohammed, O. M. Bakr, *J. Am. Chem. Soc.* **2024**, *146*, 29094.

[S19] Y. Zhang, M. Vafaie, J. Xu, J. M. Pina, P. Xia, A. M. Najarian, O. Atan, M. Imran, K. Xie, S. Hoogland, E. H. Sargent, *Adv. Mater.* **2022**, *34*, 2206884.

[S20] M. Vafaie, J. Z. Fan, A. Morteza Najarian, O. Ouellette, L. K. Sagar, K. Bertens, B. Sun, F. P. García de Arquer, E. H. Sargent, *Matter* **2021**, *4*, 1042.
